# Retinal Changes in Astrocytes and Müller Glia in a Mouse Model of Laser-Induced Glaucoma: A Time-Course Study

**DOI:** 10.3390/biomedicines10050939

**Published:** 2022-04-19

**Authors:** Jose A. Fernández-Albarral, Rosa de Hoz, José A. Matamoros, Lejing Chen, Inés López-Cuenca, Elena Salobrar-García, Lidia Sánchez-Puebla, José M. Ramírez, Alberto Triviño, Juan J. Salazar, Ana I. Ramírez

**Affiliations:** 1 Instituto de Investigaciones Oftalmológicas Ramón Castroviejo, Grupo UCM 920105, IdISSC, Universidad Complutense de Madrid, 28040 Madrid, Spain; joseaf08@ucm.es (J.A.F.-A.); rdehoz@med.ucm.es (R.d.H.); jomatamo@ucm.es (J.A.M.); lejingch@ucm.es (L.C.); inelopez@ucm.es (I.L.-C.); elenasalobrar@med.ucm.es (E.S.-G.); lidsan02@ucm.es (L.S.-P.); ramirezs@med.ucm.es (J.M.R.); atrivino@med.ucm.es (A.T.); 2 Departamento de Inmunología, Facultad de Óptica y Optometría, Oftalmología y ORL, Universidad Complutense de Madrid, 28037 Madrid, Spain; 3 Departamento de Inmunología, Facultad de Medicina, Oftalmología y ORL, Universidad Complutense de Madrid, 28040 Madrid, Spain

**Keywords:** experimental glaucoma, mice, microglia, astrocytes, Müller cell, retina, GFAP, MHC-II, ocular hypertension, contralateral eye

## Abstract

Macroglia (astrocytes and Müller glia) may play an important role in the pathogenesis of glaucoma. In a glaucoma mouse model, we studied the effects of unilateral laser-induced ocular hypertension (OHT) on macroglia in OHT and contralateral eyes at different time points after laser treatment (1, 3, 5, 8 and 15 days) using anti-GFAP and anti-MHC-II, analyzing the morphological changes, GFAP-labelled retinal area (GFAP-PA), and GFAP and MHC-II immunoreactivity intensities ((GFAP-IRI and MHC-II-IRI)). In OHT and contralateral eyes, with respect to naïve eyes, at all the time points, we found the following: (i) astrocytes with thicker somas and more secondary processes, mainly in the intermediate (IR) and peripheral retina (PR); (ii) astrocytes with low GFAP-IRI and only primary processes near the optic disc (OD); (iii) an increase in total GFAP-RA, which was higher at 3 and 5 days, except for at 15 days; (iv) an increase in GFAP-IRI in the IR and especially in the PR; (v) a decrease in GFAP-IRI near the OD, especially at 1 and 5 days; (vi) a significant increase in MHC-II-IRI, which was higher in the IR and PR; and (vii) the Müller glia were GFAP+ and MHC-II+. In conclusion, in this model of glaucoma, there is a bilateral macroglial activation maintained over time involved in the inflammatory glaucoma process.

## 1. Introduction

Glaucoma is a neurodegenerative disease of the retina characterized by the irreversible loss of retinal ganglion cells (RGCs) leading to visual loss [1,2]. Among the risk factors for this pathology, increased intraocular pressure (IOP) is one of the most important and the only one for which treatment is currently available [3,4]. However, other factors that can induce RGC death in this pathology, such as glial dysfunction, are being observed [5,6,7,8,9,10,11]. Among retinal glial cells, astrocytes and Müller glia (macroglial cells) perform functions important for neuronal activity by performing equivalent roles in the retina [12]. Müller cells are arranged radially in the retina and help to constitute the outer limiting membrane and the inner limiting membrane of the retina. They also envelop the neuronal somas and their processes, constituting an anatomical link between the neurons and the tissues with which they have to perform molecular exchanges such as the vessels, the vitreous and the subretinal space [13,14,15]. In most mammals, retinal astrocytes are mainly located in the retinal ganglion cell layer (GCL) and optic nerve fiber layer (NFL). In humans, there are two morphological types, elongated (in the NFL) and stellate in the GCL, whereas in the rat and mouse, only a plexus of stellate astrocytes arranged parallel to the retinal surface is found [12,16].

Astrocytes and Müller glia are connected by gap junctions, creating a functional syncytium involved in retinal structural organization [12]. This type of organization facilitates long-distance communication between macroglial cells [17]. They also provide physical and metabolic support to neurons and may intervene: (i) in the maintenance of extracellular ionic homeostasis, water, pH and other metabolites [18,19]; (ii) in the maintenance of the homeostasis of neurotransmitters (glutamate and GABA), which are components of the tripartite synapse [20,21,22]; (iii) in retinal glucose metabolism, storing glycogen and supplying lactate or pyruvate for oxidative metabolism [14,23]; (iv) in providing cytokines and growth factors that may produce neuroprotective or neurotoxic effects [12,24]; (v) in the elimination of toxic substances, waste products and particles potentially harmful to neurons, in addition to possessing high concentrations of antioxidants for protection against oxidative damage [25]; (vi) in the induction of blood–retinal barrier properties, ensuring the immunoprivileging of neuronal tissue [26,27,28]; (vii) in the regulation of local blood flow in responding to changes in neuronal activity [29]; (viii) in the glymphatic system, which plays a critical role in regulating the directional movement of interstitial fluids, waste removal, and central nervous system (CNS) immunity [30]; and (ix) in the immunosurveillance of the immune system as well as in the immune response [31].

When tissue damage occurs, these macroglial cells respond by a process called reactive gliosis, to defend the nerve tissue against damage and try to maintain its homeostasis [32]. However, in light of the fundamental functions mentioned above, alterations in the function of these cells could trigger damage and even neuronal death [33,34]. In reactive macrogliosis, cells undergo complex remodeling in terms of biochemistry and function [32] and undergo morphological changes characterized by the thickening of the cell body, increases in the number and length of cell processes, an increase in cell number and the upregulation of cytoskeletal components such as gliofibrillary acidic protein (GFAP), nestin and vimentin, which are considered to be the main markers of this process [8,35]. The reactivation of macroglia may initially be beneficial, as they increase their metabolic activity, increase the expression of antioxidant defenses, and restore the balance of ions, water and neurotransmitters [12,33,36]. However, if macrogliosis becomes chronic, it is harmful by directly or indirectly damaging the tissue (neurons and vessels) and preventing tissue repair [37,38,39]. Astrocytes may act in concert with microglia during the inflammatory process [40], so inflammatory mediators produced by astrocytes can chronically activate microglial cells, contributing to neuronal death [41], and similarly, inflammatory mediators released by microglia can chronically activate astrocytes [42,43]. During the neurodegenerative process, astrocytes can secrete chemokines such as CX3CL1, among others, attracting microglia, monocytes, macrophages, dendritic cells and T-cells to the site of inflammation [40]. The entry of some of these cells may be facilitated by the breakdown of the blood–retinal barrier (BRB) triggered by inflammatory mediators secreted by macroglial cells [24,44,45]. In addition, macroglial cells may express the major histocompatibility complex class II (MHC-II), acquiring the ability to act as antigen-presenting cells and stimulate T-cells [5,37].

The activation of macroglia by increased GFAP expression has been observed in human glaucoma and in animal models of glaucoma [46,47,48,49,50]. However, in the laser-induced ocular hypertension (OHT) model in rats, the decrease in the area occupied by astrocytes has been found to be even smaller with increasing IOP [8]. In this same model of glaucoma, but in mice, 15 days after OHT induction, both OHT and normotensive contralateral eyes showed a higher intensity of GFAP immunoreactivity compared to naïve eyes. Astrocytes from contralateral eyes were thicker and had a larger GFAP-labeled retinal area, but astrocytes from OHT eyes had fewer secondary processes and a smaller retinal area occupied by GFAP compared to naïve and contralateral eyes [5]. In previous work, we analyzed the behavior of microglia and the expression of cytokines and other chemokines in this glaucoma model, analyzing how it evolved at different points in time after OHT induction (1, 3, 5, 7–8 and 15 days), observing the changes at these times and finding that the major inflammatory process occurs at 3 and 5 days after OHT induction [7,9,24]. In addition, we also analyzed the number of Brn3a+ RGCs and found a progressive decrease in the number of these cells from 3 days after laser treatment in the OHT eyes [24].

Given the close relationship between the activation of macroglia and microglia and their involvement in the death of RGCs in this pathology, the present work analyzed how the activation of retinal macroglial cells occurred at different time points (1, 3, 5, 8 and 15 days) after the induction of OHT in an experimental model of laser-induced OHT in mice. For this purpose, we used retinal whole-mounts that allowed us to visualize the astroglial plexus throughout the retinal extension, analyzing the GFAP-labeled retinal area, the intensity of GFAP labeling, and the expression of MHC-II in both OHT eyes and normotensive contralateral eyes compared to naïve eyes.

## 2. Materials and Methods

### 2.1. Animals

Male Swiss albino mice aged 12–16 weeks and weighing 40–45 g were used for this work. The animals were obtained from Charles River Laboratories (Barcelona, Spain) and were subsequently kept in the animal facility of the Faculty of Medicine of the Complutense University of Madrid (Spain). The animals were monitored for temperature and light (12 h light/dark cycles and 9–24 lux). They also had free access to water and food (standard diet) ad libitum. The experiments were performed according to the ethical guidelines endorsed by Spanish law and the Guidelines for Humane Endpoints for Animals Used in Biomedical Research. The study was approved by the Ethics Committee for Animal Research of Complutense University (Madrid, Spain) and the Directorate General of Agriculture and Food, Ministry of Economy and Employment of the Community of Madrid (approval ID number: ES280790000086, 1 April 2016). The experimental procedures performed on the animals followed the institutional guidelines, European Union regulations for the use of animals in research, and the Association for Research in Vision and Ophthalmology (ARVO) statement for the use of animals in ophthalmic and vision research.

### 2.2. Experimental Groups

The mice were organized into 6 experimental groups, each consisting of 6 animals: one naïve age-matched control group and five groups of animals in which OHT was induced in the left eye and examined at 1, 3, 5, 8 and 15 days. In these groups, both the OHT eyes and their normotensive contralateral eyes were analyzed.

### 2.3. Anesthetics

The IOP was measured after sedating the animals with inhalation anesthesia using 2% isoflurane in oxygen (ISOFLO Isoflurane 100% p/p, Zoetis SL, Alcobendas, Madrid, Spain). OHT was induced under general anesthesia using an intraperitoneal injection of a mixture of ketamine (75 mg/kg; Anesketin^®^, Dechra Veterinary Products SLU, Barcelona, Spain) and medetomidine (0.26 mg/kg; Medetor^®^, Virbac España S.A., Barcelona, Spain). To prevent corneal desiccation and infection after the laser procedure, tobramycin (Tobrex^®^; Alcon, Barcelona, Spain) ointment was used. After OHT induction, all the appropriate procedures were adopted to mitigate the pain and discomfort of the animals. The animals were finally sacrificed after the intraperitoneal injection of an overdose of pentobarbital (Dolethal Vetoquinol^®^, Especialidades Veterinarias, Alcobendas, Madrid, Spain).

### 2.4. Laser Treatment and Measurement of IOP

OHT was induced by a single session of diode laser burns (Viridis Ophthalmic Photocoagulator-532 nm, Quantel Medical, Clermont-Ferrand, France) in the left eyes of previously anesthetized animals, using a previously described method [51,52]. The laser burns (55–76) were performed by applying the laser beam directly on the episcleral and limbal veins using the following parameters: spot size, 50–100 μm; duration, 0.5 s; and power, 0.3 W. In both the laser-treated and contralateral eyes, the IOP was measured using a rebound tonometer (Tono-Lab, Tiolat, OY, Helsinki, Finland) [53] in all the animals of the different study groups. Six consecutive measurements were taken and averaged. The IOP measurements were taken after anesthetizing the animal and preferably at the same time of day (9 a.m.) to avoid variations due to circadian rhythms [53]. The IOP was measured before animal sacrifice in the eyes of the naïve group. In the eyes (OHT and contralateral) of the five groups in which OHT was induced, IOP was measured before laser treatment and at 1, 3, 5, 8 and 15 days after laser treatment.

### 2.5. Immunohistochemistry

Transcardiac perfusion was performed to fix the animals. First, a saline wash solution was introduced to eliminate blood, and then, a fixative solution consisting of 4% paraformaldehyde in 0.1 M phosphate buffer (PB) at pH 7.4 was perfused. A stitch was made on the upper eyelid as a marker to help to maintain the orientation of the eyeball. The caruncle and rectus muscles were also used as complementary markers for orientation. The eyes were extracted and postfixed in 4% paraformaldehyde in BP (pH 7.4, 0.1 M) for 2 h. After this, the retinas were extracted and whole-mounts were made [8]. In the retinas, double immunofluorescences were induced using anti-GFAP, to label the macroglial cells (astrocytes and activated Müller glia), to analyze the effects of OHT on retinal macroglia and MHC-II, and to check whether astrocytes and Müller glia could acquire antigen-presenting-cell characteristics after OHT induction. Retinas were incubated with polyclonal anti-GFAP chicken, 1:100 (ref. AB5541, Millipore, Massachusetts, MA, USA), and anti-MHC-II mouse developed in rat, 1:100 (ref. 14-5321-82, eBioscience, San Diego, CA, USA), antibodies. The respective secondary antibodies used were donkey anti-chicken antibody conjugated with DyLight™ 405, 1:100 (ref. 703-475-155, Jackson Immunoresearch, West Grove, PA, USA), and goat anti-rat Alexa Fluor 488, 1:150 (ref A21208, Invitrogen, Paisley, UK). The following negative controls were performed: In the first, the primary antibodies were not added, and the retinal tissue was incubated only in the secondary antibodies with their respective diluents. In the second, the tissue was incubated in the primary antibodies with their diluents without adding secondary antibodies. In the third, only the diluents of the primary and secondary antibodies were added, to observe the endogenous fluorescence provided by the tissue. The retinas were then analyzed by fluorescence microscopy (Zeiss Axio Imager M.2, Carl Zeiss AG, Oberkochen, Germany) with a microscope equipped with an ApoTome device (Apotome-2 module, Carl Zeiss AG, Oberkochen, Germany) and a high-resolution digital camera (Axio Cam 503 Mono (Carl Zeiss AG, Oberkochen, Germany)). For the visualization of the Alexa Fluor 488 and the DyLight™ 405, the microscope was equipped with a Zeiss 10 filter set and Filter Set 49, Zeiss, respectively. For the study of the retinas, retinal whole-mounts were analyzed along the xyz axes using the motorized microscope stage, acquiring images with the apotome device. The apotome creates optical sections of fluorescent samples, free of stray light, and increases the resolution in the Z-direction compared to conventional fluorescence microscopy. The Z stacks were analyzed in the ZEN2 software (Carl Zeiss AG, Oberkochen, Germany).

Adobe Photoshop CS6 Extended 10.0 (Adobe Systems, San José, CA, USA) was used to assemble the plates of the images of the figures. For a better visualization of the tissues labeled with the GFAP–DyLight™ 405 in the images of the plates, the blue color was changed to green or red.

### 2.6. Quantitative Retinal Analysis

To determine the effect exerted by OHT on the retinal macroglia at the different study time points (1, 3, 5, 8 and 15 d), the following parameters were quantified in a double-blind fashion: the GFAP-labeled retinal area (GFAP-RA), intensity of GFAP immunoreactivity (GFAP-IRI) and intensity of MHC-II immunoreactivity (MHC-II-IRI).

#### 2.6.1. GFAP-RA

To quantify the GFAP-RA in the retinal whole-mounts, photographs were taken in the left eyes of all the animals in the naïve group and in both the OHT eyes and their normotensive contralateral eyes of all the animals in the five groups of the laser-treated study groups.

Two different metrics were used to quantify GFAP-RA: (i) the total retinal area occupied by GFAP immunostaining and (ii) sectoral analysis within the four quadrants of the retina for GFAP immunolabeling. For the total area of GFAP-RA, each entire retinal whole-mount was analyzed using the automatic field displacement tool of the motorized stage of the microscope to scan its entire length along the X–Y axis. Thus, all the analyzed fields were contiguous and were photographed systematically to prevent any portion of the retinal whole-mount being omitted or duplicated, thereby analyzing about 1200 fields from the six study groups (six animals per group).

For sectorial quantification, photographs of three equivalent areas (the near optic disc (OD), intermediate retina (IR) and peripheral retina (PR)) of the vertical and horizontal meridians that cross the optic nerve and include the superior (S), inferior (I), nasal (N) and temporal (T) retinal areas were taken. Twelve fields per retina were analyzed; as six animals were studied in each group, a total of 72 fields were measured (in the laser groups: 72 for the OHT eyes and 72 for the contralateral eyes); all the groups together accounted for a total of 792 fields examined. All the photographs were taken by analyzing the retina at 20× magnification, which provided an area of 0.1502 mm^2^ per field.

To quantify GFAP-RA, a threshold tool in MATLAB was used on the microphotographs taken. The thresholds determine the pixels of the objects of interest based on the grayscale values, allowing them to be differentiated from other areas of the image based on the grayscale values of the images. The GFAP-RA was then quantified using an algorithm developed by our group in the MATLAB environment [54].

#### 2.6.2. GFAP-IRI and MHC-II-IRI

To quantify the GFAP-IRI and MHC-II-IRI of the retinal whole-mounts, photographs were taken at 20× for GFAP-IRI and 40× for MHC-II-IRI. For each of the GFAP-IRI or MHC-II-IRI quantifications, all the photographs were taken with equal values of exposure time and excitation intensity, to allow qualitative comparisons. As with the GFAP-RA, photographs were taken of three equivalent areas of the retina (near the optic nerve head, intermediate retina and peripheral retina) in the vertical and horizontal meridians that included the superior, inferior, nasal and temporal sectors of the retina. To quantify the GFAP and MHC-II expression intensity, images were analyzed with the ImageJ software (developed by Wayne Rasband, National Institutes of Health, Bethesda, MD, USA; https://imagej.nih.gov/ij/ (accessed on 8 January 2022). The quantification protocol in ImageJ was performed based on the work of Schindelin et al., 2012 [55]. The images were converted to 8-bit format and adjusted based on a threshold. The average values measured using the ImageJ tool were the result of normalizing the average values of the image intensity by 255, which is the maximum value that each pixel can represent in 8-bit images.

Schematic representations of the retinal whole-mount with the retinal sectors analyzed in the four quadrants were used to show the results. All the schematic retinas show statistically significant differences and have the same predefined color scale that assigns a color to each mean value of occupied retinal area, highlighting different levels of GFAP-RA, GFAP-IRI and MHC-II-IRI, from 0 to the maximum measurement obtained.

### 2.7. Statistical Analysis

Statistical analysis was performed with SPSS 25 (IBM, Armonk, NY, USA). The data are presented as the means ± SDs. The following parameters were analyzed—IOP, GFAP-RA, GFAP-IRI and MHC-II-IRI—using the Wilcoxon W test (for paired data) and Mann–Whitney U test (for unpaired data). The differences between contralateral eyes and OHT eyes were analyzed by ANOVA tests with Bonferroni correction, at the different time points of the study. Differences were considered statistically significant when *p* < 0.05.

## 3. Results

### 3.1. Intraocular Pressure (IOP)

In the OHT-induced eyes, there was an increase in IOP that was maximal with respect to naïve eyes at 1 and 3 d after laser treatment (both *p* < 0.01; Supplementary Figure S1). The IOP gradually began to decrease at 5 days; although, it remained higher than that in naïve eyes (*p* < 0.01; Supplementary Figure S1). However, at 8 days, the IOP of the OHT-induced eyes was similar to that of the naïve eyes. The contralateral eyes of animals from all the time points of the study (1, 3, 5, 8 and 15 days) had IOPs similar to those of naïve eyes (Supplementary Figure S1).

### 3.2. General Characteristics of Astrocytes and Müller Glia in the Naïve Retina

**Figure 1:**
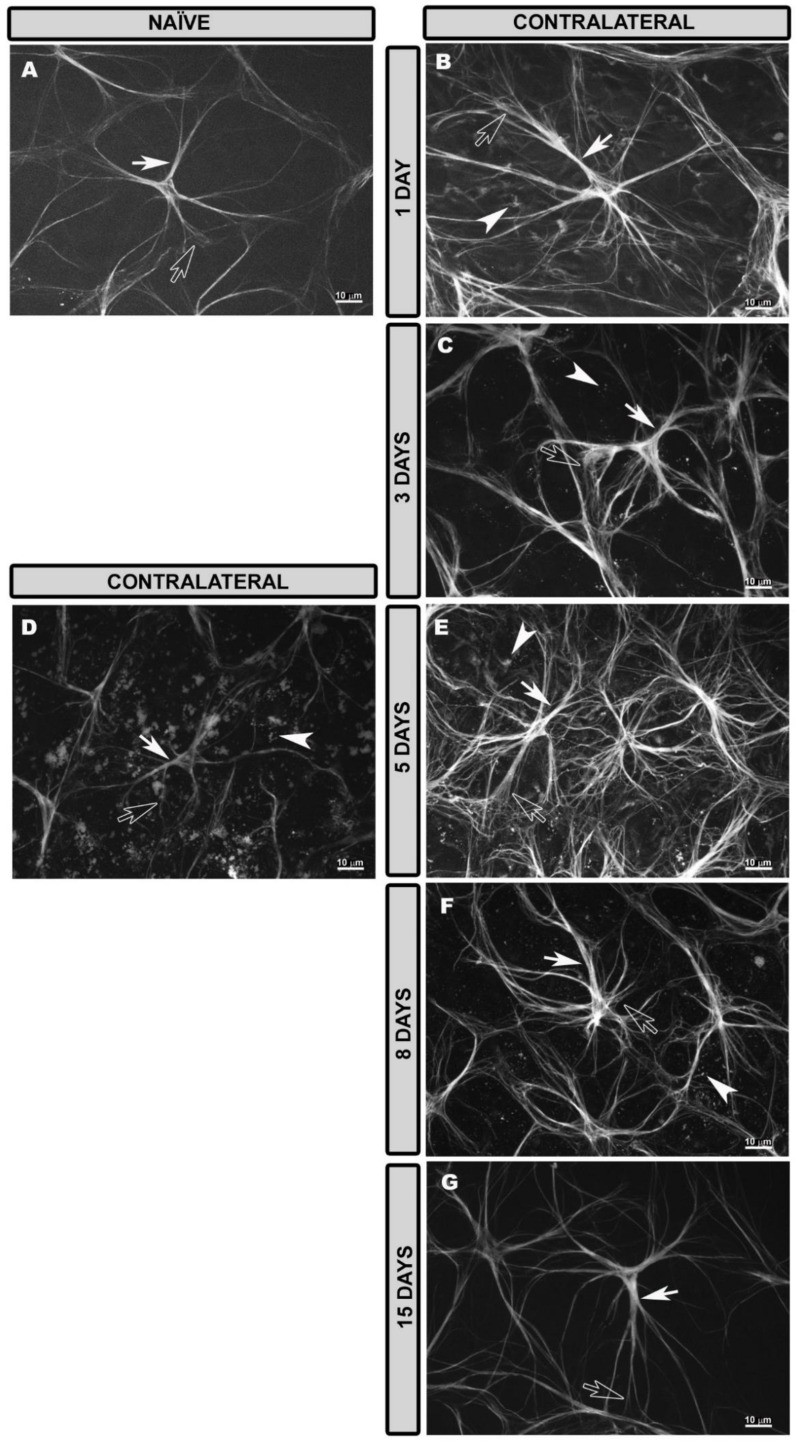
Retinal whole-mount. GFAP immunostaining in naïve eyes and at different time points after OHT induction in contralateral eyes (high magnification). Morphological characteristics of macroglial cells in naïve eyes (**A**) and contralateral eyes (**B**–**G**) at 1, 3, 5, 8 and 15 days after laser treatment. In naïve retinas, astrocytes had stellate shapes with rounded cell bodies and several primary (white arrow) and secondary (hollow arrow) processes (**A**). In contralateral retinas at 1 (**B**), 3 (**C**) and 8 (**F**) days after laser treatment, astrocytes were reactive with thick somas and numerous primary (white arrow) and secondary (hollow arrow) processes. At 5 days, astrocytes were highly reactive, with a large number of secondary processes (hollow arrow) (**E**). In areas close to the optic disc, astrocytes with low GFAP intensity were observed, and secondary processes were difficult to observe (**D**). At 15 days after laser treatment, astrocytes were slightly more reactive (**G**) than naïve. The end feet (arrowhead) of activated Müller glia were visualized at 1 (**B**), 3 (**C**), 5 (**E**) and 8 (**F**) days after laser induction. GFAP: glial fibrillary acidic protein; OHT: ocular hypertension.

In naïve eyes, GFAP+ astrocytes had rounded cell bodies from which numerous primary and secondary processes extended radially, giving the cells a stellate appearance (Figure 1A and Figure S2A,B). The astrocytes formed a honeycomb plexus that was regularly distributed from the optic disc to the peripheral retina at the level of the NFL-GCL (Supplementary Figure S3A,D,G). In the plexus, the cells were easily distinguishable from each other (Supplementary Figures S2A,B and S3A,D,G). Astrocytes demarcated the blood vessels with both their somas and processes and clearly followed the course of the vessels from the optic disc (Supplementary Figure S3A). The circular vessel surrounding the peripheral retina was also clearly visualized because of the numerous astrocytes arranged in the vessel wall (Supplementary Figure S3G). Müller glia were generally not visualized by GFAP immunostaining; only occasionally were GFAP+ punctate structures observed in the peripheral retina, which would correspond to the terminal end feet of Müller glia. MHC-II expression was not observed in either astrocytes or Müller glia, only in dendritic cells and perivascular microglia (Supplementary Figure S3B,C,E,F,H,I).

### 3.3. Morphological Changes in Retinal Macroglia of Contralateral Eyes at Different Time Points after OHT Induction

At higher magnification, it was observed that, 1 day after laser treatment, the astrocytes had a stellate appearance (Figure 1B) and were more reactive than the naïve astrocytes, as they had more robust cell bodies with thicker and more numerous processes (Figure 1B). At 3 days after the laser treatment, the astrocytes showed similar characteristics to the 1-day contralateral ones; although, they were slightly more reactive (thicker and with more processes) (Figure 1C). At 5 days after the laser treatment in the retina near the optic disc, some astrocytes appeared to have lower GFAP intensity and only their primary processes were observed with secondary processes disappearing, all of which made the cells appear more diffuse (Figure 1D). However, in the intermediate and peripheral retina, the astrocytes were more reactive than at 3 days contralateral, being thicker, with more processes and with an apparent higher GFAP intensity (Figure 1E). At 8 days after laser treatment, reactive astrocytes (Figure 1F) were observed throughout the retina similar to those in the contralateral eyes 3 days after treatment. At 15 days, the astrocytes were slightly more reactive (Figure 1G) than the naïve, but less so than the contralateral at 1, 3, 5 and 8 days (less thickened astrocytes with fewer secondary processes).

In contralateral eyes as in naïve eyes, astrocytes formed a plexus from the optic disc (OD) to the peripheral retina (PR). This plexus in the areas near the OD was slightly denser than that in the naïve at all the time points (1, 3, 5, 8 and 15 days) after laser treatment (Supplementary Figure S4A,D,G,J,M).

**Figure 2:**
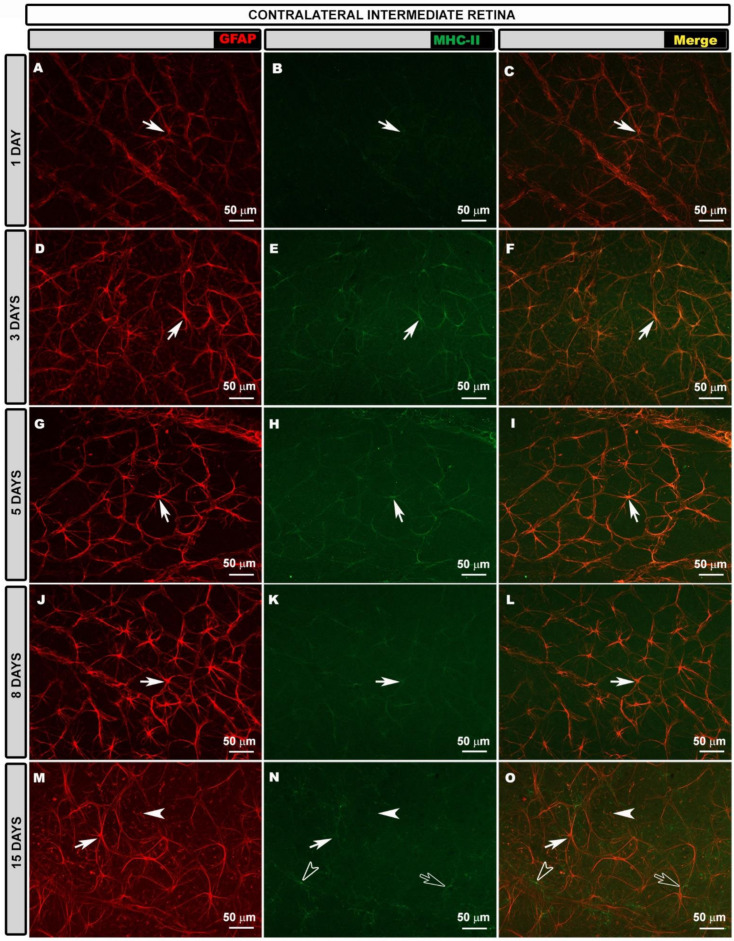
Retinal whole-mount. Double immunostaining for GFAP (red) and MHC-II (green) at different time points after OHT induction in contralateral eyes in the intermediate retina. GFAP-labeled astrocytes are shown at different time points (**A**,**D**,**G**,**J**,**M**). In the intermediate retina of contralateral eyes, MHC-II expression by astrocytes (arrow) was increased relative to that in areas near the OD at all time points (1 (**B**,**C**); 3 (**E**,**F**); 5 (**H**,**I**); 8 (**K**,**L**); and 15 (**N**,**O**) days) after OHT induction. At 15 days, microglia (hollow arrow) and end feet of Müller glia (arrowhead) also showed MHC-II immunolabeling. Images show perivascular microglia (hollow arrowhead) with high MHC-II expression (**N**,**O**). GFAP: glial fibrillary acidic protein; MHC-II: major histocompatibility complex class II; OHT: ocular hypertension; OD: optic disc.

In the intermediate retina (IR) of the contralateral eyes, the astroglial plexus was denser than in the naïve at all the time points after laser treatment (Figure 2A,D,G,J,M), but it was much denser at 3 (Figure 2D), 5 (Figure 2G) and 8 (Figure 2J) days because, at these time points, the astrocytes were much more reactive in this retinal area.

**Figure 3:**
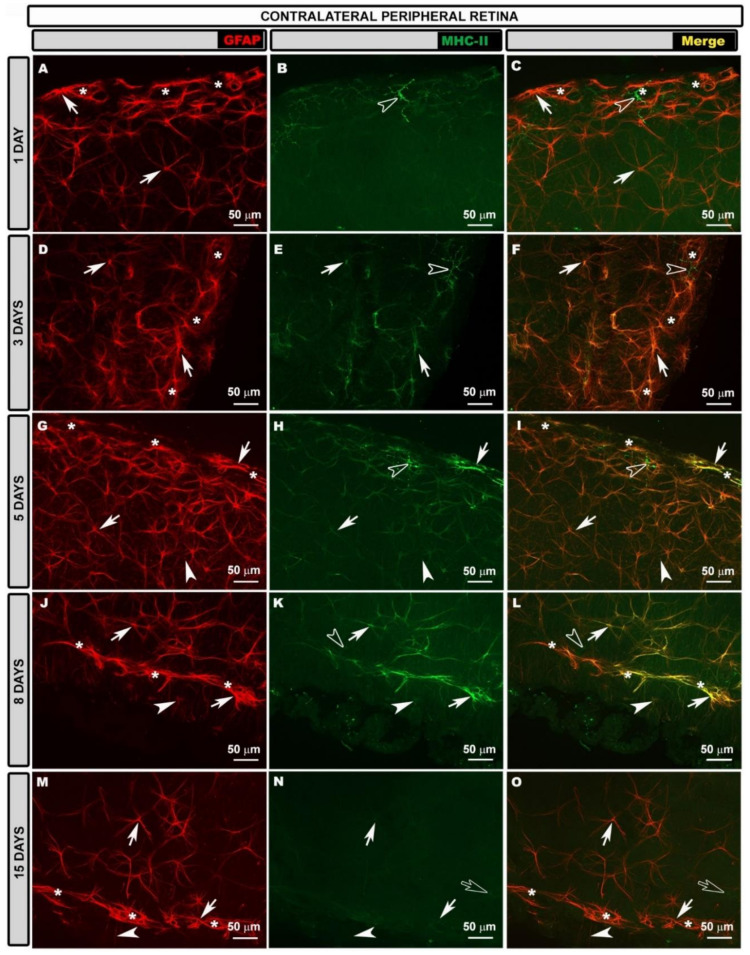
Retinal whole-mount. Double immunostaining for GFAP (red) and MHC-II (green) at different time points after OHT induction in contralateral eyes in the peripheral retina. GFAP-labeled astrocytes are shown at different time points (**A**,**D**,**G**,**J**,**M**). In the peripheral retina, astrocytes (arrow) showed intense MHC-II expression that was comparable to that of dendritic cells (hollow arrowhead) at all time points (1 (**B**,**C**); 3 (**E**,**F**); 5 (**H**,**I**); and 8 (**K**,**L**) days) after laser tr.eatment except at 15 (**N**,**O**) days, at which time microglia (hollow arrow) also expressed MHC-II. MHC-II immunolabeling in Müller glia (arrowhead) was observed at 5 (**H**,**I**); 8 (**K**,**L**); and 15 (**N**,**O**) days. Asterisks mark circular peripheral blood vessel. GFAP: glial fibrillary acidic protein; MHC-II: major histocompatibility complex class II; OHT: ocular hypertension.

In the PR, the highly reactive astrocytes formed a very dense plexus at 1 (Figure 3A), 3 (Figure 3D), 5 (Figure 3G) and 8 (Figure 3J) days after laser treatment. However, 15 days after laser treatment (Figure 3M), the plexus was less dense than at the previous time points, but denser than for the naïve eyes. At all the time points after laser treatment, vascular sheaths formed by reactive astrocytes were observed in the circular vessel of the peripheral retina.

Regarding MHC-II expression, contrary to what occurred in the naïve eyes, astrocytes in the contralateral eyes expressed MHC-II at 1, 3, 5 and 8 days after laser treatment (Figure 2, Figure 3 and Figure S4). This expression was less intense in the areas near to the OD at all the time points (Supplementary Figure S4B,C,E,F,H,I,K,L,N,O) and increased in the IR (Figure 2B,C,E,F,H,I,K,L,N,O), being maximal in the PR (Figure 3B,C,E,F,H,I,K,L,N,O), especially at 3, 5 and 8 days after laser treatment, as intensely as in dendritic cells. At 15 days after laser treatment, microglial cells also expressed MHC-II, and astrocytes near to the OD did not express MHC-II (Supplementary Figure S4N,O), while IR and PR astrocytes had medium or low MHC-II expression (Figures 2N,O and 3N,O).

**Figure 4:**
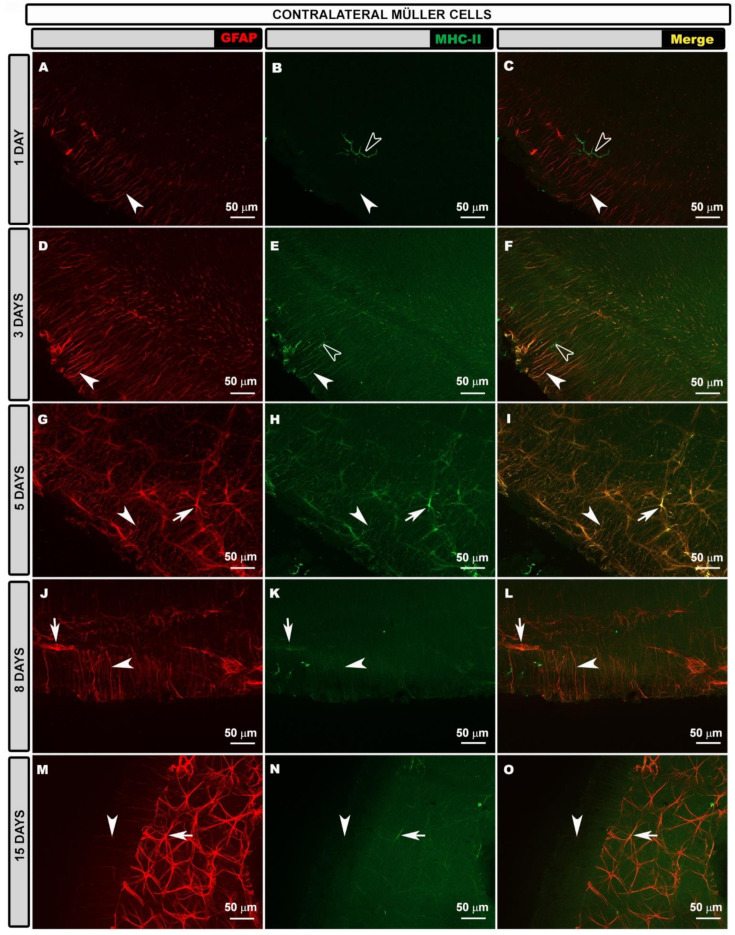
Retinal whole-mount. Double immunostaining for GFAP (red) and MHC-II (green) in Müller glia at different time points after OHT induction in contralateral eyes. GFAP-labeled Müller cells are shown at different time points (**A**,**D**,**G**,**J**,**M**). MHC-II expression by Müller glia (arrowhead) was observed at all time points (1 (**B**,**C**); 3 (**E**,**F**); 5 (**H**,**I**); 8 (**K**,**L**); and 15 (**N**,**O**) days) after laser treatment, being more intense at 3 (**E**,**F**) and 5 (**H**,**I**) days when the expression was comparable to that for dendritic cells (hollow arrowhead). Arrows point to MHC-II+ astrocytes. GFAP: glial fibrillary acidic protein; MHC-II: major histocompatibility complex class II; OHT: ocular hypertension.

In contrast to those in naïve eyes, the Müller glia in contralateral eyes were reactive, as they expressed GFAP. The GFAP+ end feet of these cells were observed along the retinal surface at all the time points after laser treatment (Supplementary Figure S2C,E,G,I,K). When analyzing the edge of the preparation where flattening the tissue provides us with the image of a histological section, we could observe that the Müller glia were GFAP+ in all their extensions at all the time points analyzed after laser treatment (Figure 4A,D,G,J,M). However, the GFAP+ immunoreaction was greater at 3 (Figure 4D), 5 (Figure 4G) and 8 (Figure 4J) days. At 15 days after laser treatment, Müller cells were reactive but their GFAP expression was apparently lower than that at earlier time points for the contralateral eyes (Figure 4M).

The MHC-II expression in the Müller glia was low at 1 day (Figure 4B,C) after laser treatment, increasing at 3 (Figure 4E,F) and 5 days (Figure 4H,I), and decreasing again at 8 (Figure 4K,L) and 15 days (Figure 4N,O).

### 3.4. Morphological Changes in Retinal Macroglia of OHT Eyes at Different Time Points after OHT Induction

**Figure 5:**
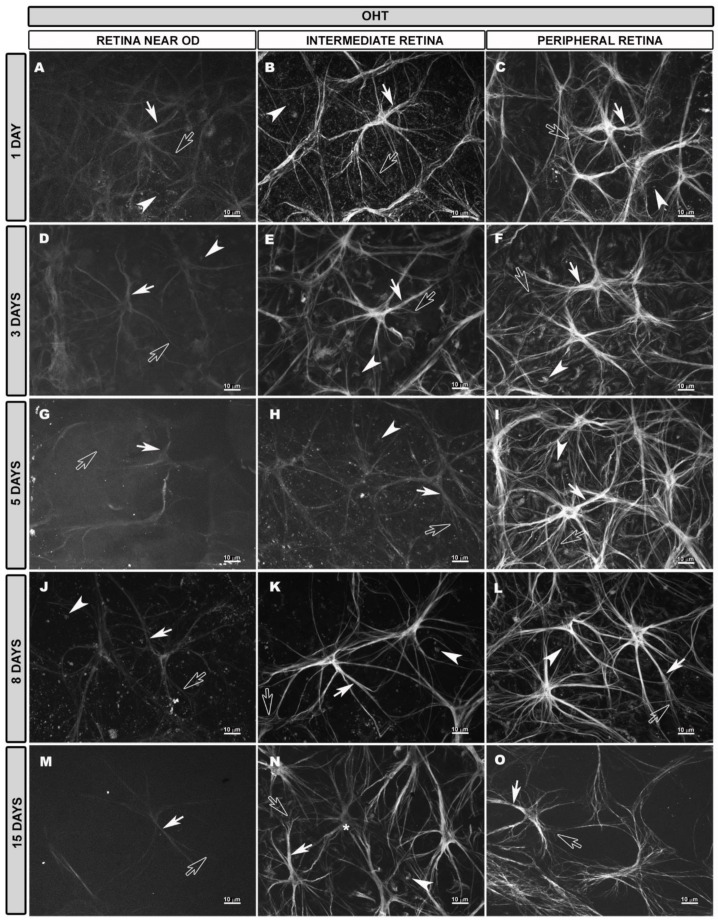
Retinal whole-mount. GFAP immunostaining at different times after OHT induction in eyes with OHT (high magnification). Morphological characteristics of macroglial cells in eyes with OHT at 1, 3, 5, 5, 8 and 15 days after laser treatment (**A**–**O**). Astrocytes near the optic disc (OD) had low GFAP immunoreactivity, and only soma and primary processes (white arrow) were visualized at 1 (**A**), 3 (**D**), 5 (**G**) 8 (**J**) and 15 (**M**) days after laser treatment. In the intermediate retina, astrocytes were more reactive than those in naïve, with thicker primary processes (white arrow) and more numerous secondary processes (hollow arrow) at 1 (**B**), 3 (**E**) and 8 (**K**) days after laser treatment. At 5 days, in this area, astrocytes with low GFAP immunoreactivity were observed in which secondary processes were difficult to visualize (hollow arrow) (**H**). At 15 days after laser treatment, astrocytes with low reactivity (asterisk) alternated with astrocytes with high reactivity (**N**). In the peripheral retina, astrocytes were highly reactive, with thick somas and primary processes (white arrow) and numerous secondary processes (hollow arrow) at 1 (**C**), 3 (**F**), 5 (**I**) and 8 (**L**) days after laser treatment. At 15 days, astrocytes were less reactive (**O**) than at previous time points. Throughout the retina, end feet of activated Müller glia (arrowhead) were visualized (**A**–**F**,**H**–**L**,**N**). GFAP: glial fibrillary acidic protein; OHT: ocular hypertension; OD: optic disc.

At higher magnification, it was observed that, 1 day after OHT induction, in areas near to the OD, the astrocytes were slightly thicker than those in naïve eyes and were cells in which the secondary processes disappeared. These cells had an apparently lower intensity of GFAP than naïve and contralateral eyes (Figure 5A). In the IR, the astrocytes were slightly thicker and with more secondary processes (Figure 5B and Figure S5D) than in the naïve group. The astrocytes in the PR showed a higher state of activation, being thicker, with abundant processes and an apparent higher GFAP+ intensity (Figure 5C) compared to those in the other regions and the naïve group. At 3 days after laser treatment, close to the OD, astrocytes with low GFAP+ immunoreactivity and few secondary processes (Figure 5D) similar to the OD astrocytes for 1-day OHT eyes were observed. In the IR, the astrocytes were reactive (Figure 5E and Figure S5F), apparently similar to those at 1 day. In the PR, the astrocytes were highly reactive and intensely labeled with GFAP, with thick somas and abundant primary and secondary processes (Figure 5F), as occurred for the 1-day OHT eyes. At 5 days after laser treatment, in the areas close to the OD, it was difficult to visualize astrocytes because only some somas and primary processes were differentiated and the GFAP+ immunoreactivity of the astrocytes was even lower than at 1 and 3 days (Figure 5G). For the 5-day OHT eyes, in the IR, the astrocytes were more difficult to visualize than those for the 3-day OHT eyes, due to their low GFAP+ staining and poor visualization of their processes (Figure 5H and Figure S5H). As occurred for the 1- and 3-day OHT eyes in the PR, the astrocytes were very reactive (Figure 5I). For the 8-day OHT eyes, near to the OD, the astrocytes were more easily observed than those for the 3- and 5-day OHT eyes; although, they showed a lower GFAP intensity and the disappearance of secondary processes (Figure 5J) as for the 1-day OHT eyes. In the IR, the astrocytes were reactive (thicker, with more secondary processes) (Figure 5K), as occurred for the 1-day OHT eyes. In the peripheral retina, for the 8-day OHT eyes, as at Days 1, 3 and 5, the astrocytes were highly reactive (Figure 5L). At 15 days after OHT induction, in the areas near to the OD, the astrocytes were similar to those for the 1-day and 8-day OHT eyes (Figure 5M). In the IR, astrocytes with only primary processes and low GFAP intensity, alternating with reactive astrocytes (thicker, with more processes and high GFAP intensity), were observed (Figure 5N and Figure S5L). In the PR, the astrocytes were apparently less reactive than those at the previous time points (Figure 5O).

In OHT eyes, the astrocytes formed a plexus as in naïve eyes, but their better or worse visualization depended on both the retinal area and the time point after laser treatment.

At the level of the OD, at all the time points after laser treatment, the honeycomb plexus was more difficult to visualize due to the decreased GFAP+ immunoreactivity of astrocytes (Supplementary Figure S5A,D,G,J,M). The vessels originating from the OD had a blurred appearance because the perivascular astrocytes appeared as lines, since part of their processes had disappeared, and it was difficult to visualize their soma (Supplementary Figure S5A,D,G,J,M).

**Figure 6:**
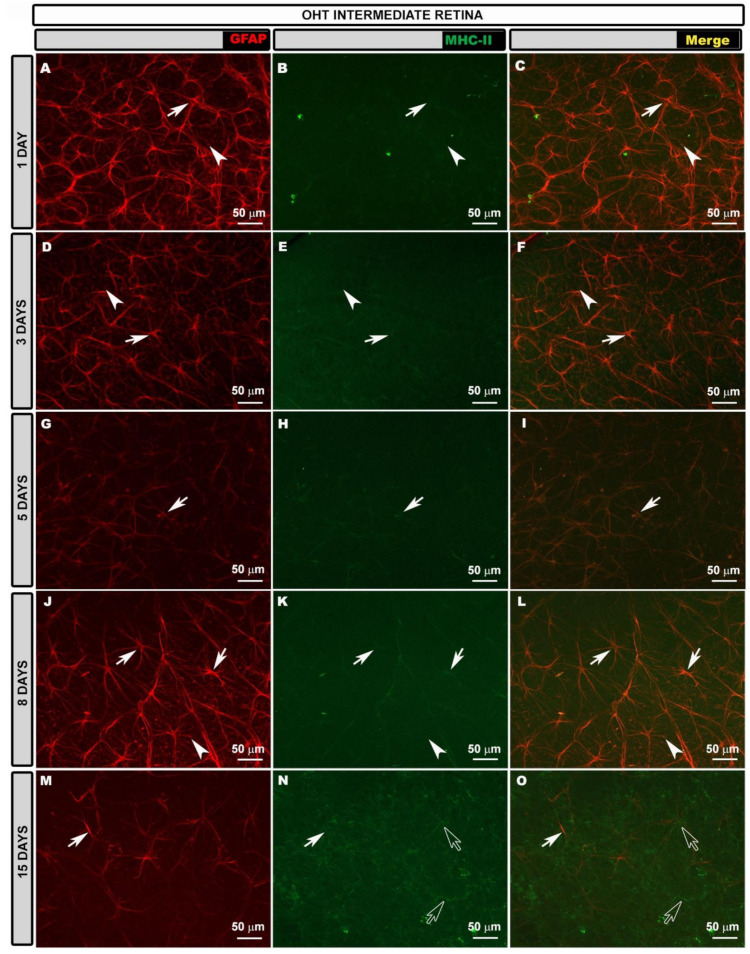
Retinal whole-mount. Double immunostaining for GFAP (red) and MHC-II (green) at different time points after OHT induction in OHT eyes in the intermediate retina. GFAP-labeled astrocytes are shown at different time points (**A**,**D**,**G**,**J**,**M**). In the intermediate retina, MHC-II expression by astrocytes (arrow) was slightly higher than that found in the retina near to the OD at all time points (1 (**B**,**C**); 3 (**E**,**F**); 5 (**H**,**I**); 8 (**K**,**L**); and 15 (**N**,**O**) days) after OHT induction. At 15 days after laser treatment (**N**,**O**), intense MHC-II expression by microglia (hollow arrowhead) was observed. The arrowhead points to the terminal feet of Müller glia. GFAP: glial fibrillary acidic protein; MHC-II: major histocompatibility complex class II; OHT: ocular hypertension; OD: optic disc.

In the IR, the astroglial plexus was denser than that in the naïve eyes at 1 and 8 days after laser treatment (Figure 6A,J) because the astrocytes were much more reactive. However, at 3 and 5 days after laser (Figure 6D,G), the plexus was less well visualized because some astrocytes showed decreased GFAP+ immunoreactivity, and Müller glia showed increased GFAP expression. At 15 days after laser treatment (Figure 6M), the astroglial plexus was more similar to the naïve one.

**Figure 7:**
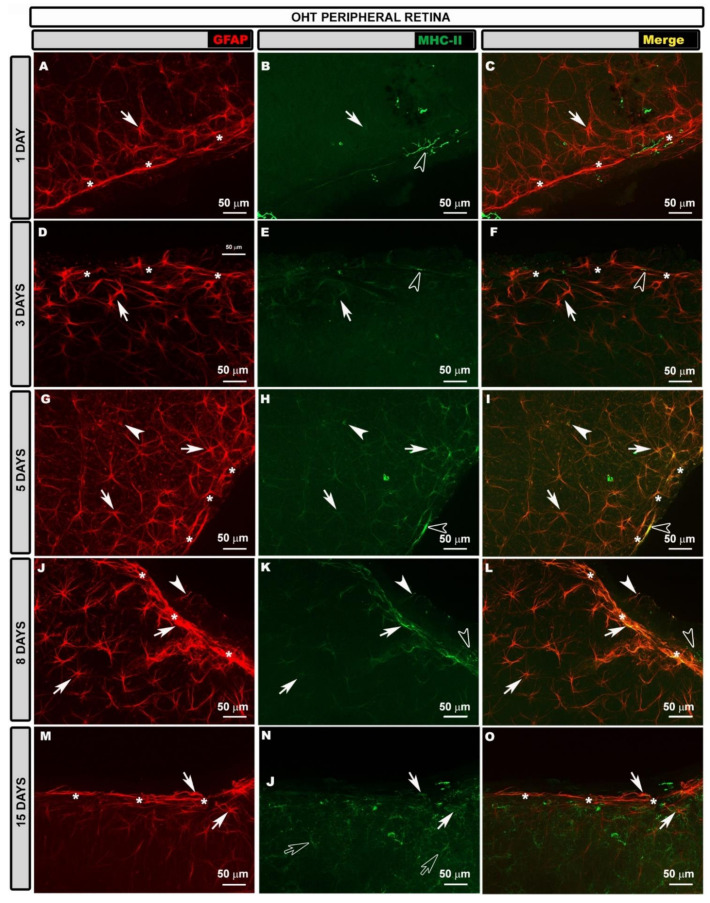
Retinal whole-mount. Double immunostaining for GFAP (red) and MHC-II (green) at different time points after OHT induction in OHT eyes in the peripheral retina. GFAP-labeled astrocytes are shown at different time points (**A**,**D**,**G**,**J**,**M**). In the peripheral retina, the expression of MHC-II by astrocytes (arrow) was more intense than in the intermediate retina at all time points (1 (**B**,**C**); 3 (**E**,**F**); 5 (**H**,**I**); 8 (**K**,**L**); and 15 (**N**,**O**) days), being comparable to that of dendritic cells (hollow arrowhead). At 15 days, microglial cells (hollow arrow) expressed MHC-II. The arrowhead points to the terminal feet of Müller glia. Asterisks mark circular peripheral blood vessel. GFAP: glial fibrillary acidic protein; MHC-II: major histocompatibility complex class II; OHT: ocular hypertension.

In the PR, at 1, 3, 5 and 8 days after OHT induction (Figure 7A,D,G,J), the astrocytes, which were highly reactive, formed a very dense plexus. However, at 15 days (Figure 7M), although the plexus was less dense than at previous time points, it was denser than in the naïve eyes. As occurred in the contralateral eyes, vascular sheaths formed by reactive astrocytes were observed in the circular vessel of the peripheral retina at all the time points of the study (Figure 7A,D,G,J).

Regarding the MHC-II expression in OHT eyes, we observed different patterns of expression of this molecule in astrocytes depending on the retinal area and the time point after the laser treatment. At the OD, 1 day after OHT induction (Supplementary Figure S5B,C), no MHC-II expression was observed in astrocytes. At later time points (3, 5, 8 and 15 days) (Supplementary Figure S5E,F,H,I,K,L,N,O), astrocyte MHC-II expression was very low. At 15 days (Supplementary Figure S5N,O), the main MHC-II expression was by microglia. IR astrocytes showed low MHC-II expression at all the time points analyzed (Figure 6B,C,E,F,H,I,K,L,N,O). In the PR, the expression of MHC-II by astrocytes increased considerably at all the time points except at 15 days (Figure 7B,C,E,F,H,I,K,L,N,O), being similar in many cases to that of dendritic cells. At 15 days after laser treatment (Figure 7N,O), the MHC-II expression by astrocytes was low, as in the rest of the regions.

**Figure 8:**
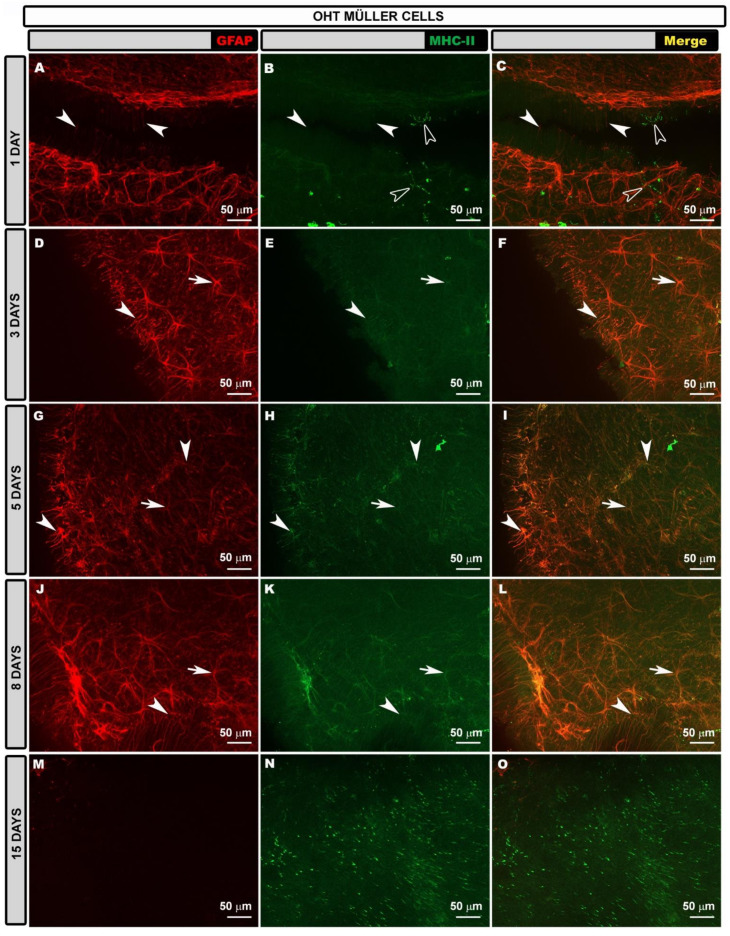
Retinal whole-mount. Double immunostaining for GFAP (red) and MHC-II (green) in Müller glia at different time points after OHT induction in OHT eyes. GFAP-labeled Müller cells are shown at different time points (**A**,**D**,**G**,**J**,**M**). MHC-II expression in Müller glia (arrow head) was observed at all time points (1 (**B**,**C**); 3 (**E**,**F**); 5 (**H**,**I**); 8 (**K**,**L**); and 15 (**N**,**O**) days), being more intense at 15 (**N**,**O**) days after laser treatment, being as intense as that of dendritic cells (hollow arrowhead). Arrows point to MHC-II+ astrocytes. GFAP: glial fibrillary acidic protein; MHC-II: major histocompatibility complex class II; OHT: ocular hypertension.

In OHT eyes, as in contralateral eyes, Müller glia were reactive throughout the retina and their end feet were observed along the retinal surface at all the time points after laser treatment (Supplementary Figure S2D,F,H,J,L). When the edges of the preparation were analyzed, the entire extent of the Müller glia was GFAP+ (Figure 8A,D,G,J,M), and this was observed at all the time points analyzed, being more intense at 3, 5 and 8 days after the laser treatment (Figure 8D,G,J) and less intense at 15 days (Figure 8M). At 5 and 8 days after laser treatment in the PR, there were areas of glial scars formed by Müller cells and astrocytes (Supplementary Figure S6).

The MHC-II expression in the Müller glia differed according to the time point after laser treatment. At 1 and 3 days after laser treatment (Figure 8B,C,E,F), the expression was very low, increasing after 5 days (Figure 8H,I) and being higher at 15 days (Figure 8N,O). Interestingly, at 15 days after laser treatment, the higher expression of MHC-II by Müller cells coincided with a lower expression of GFAP. Additionally, at this time point, the MHC-II expression was higher in Müller glia than in astrocytes.

### 3.5. GFAP-Labeled Retinal Area at Different Time Points after OHT Induction

#### 3.5.1. GFAP-RA Total Value

**Figure 9:**
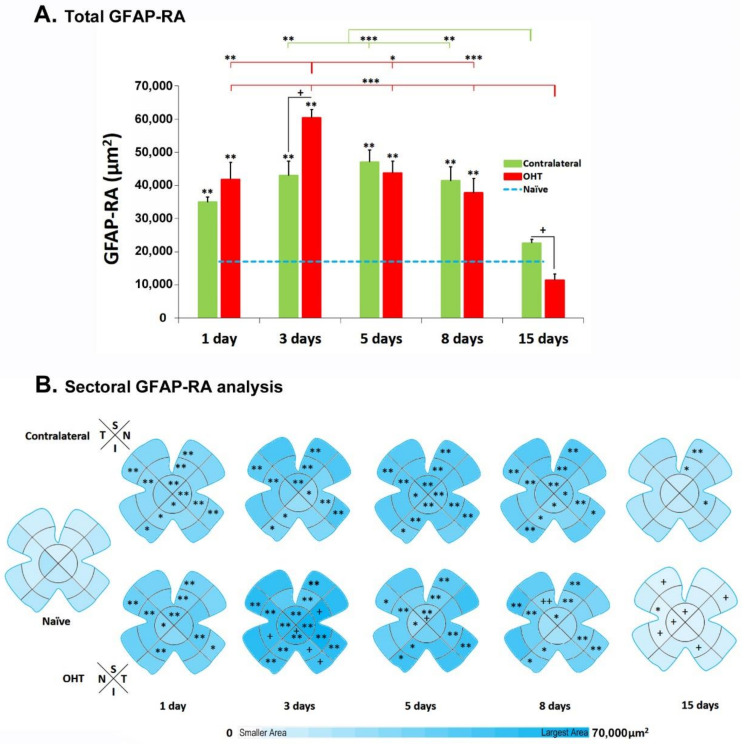
GFAP-RA values at different time points after unilateral OHT laser induction. (**A**): Histogram shows the mean values of total GFAP-RA (±SD) at different time points after laser treatment (1, 3, 5, 8 and 15 days) in contralateral and OHT eyes. Dashed line represents GFAP-RA measurements in naïve eyes. The GFAP-RA total increase at 1 day remained statistically elevated until 8 days, being more intense at 3 and 5 days after laser in both OHT eyes and contralateral eyes. ** *p* < 0.01 OHT vs. naïve and contralateral vs. naïve; Mann–Whitney U test. + *p* < 0.05 OHT vs. contralateral; Wilcoxon W test. * *p* < 0.05, ** *p* < 0.01, *** *p* < 0.001 OHT and contralateral variation over time; ANOVA Bonferroni test. (**B**): Colorimetric differences in the GFAP-RA by retinal sector in naïve eyes and at different time points after laser treatment (1, 3, 5, 8 and 15 days) in contralateral and OHT eyes. * *p* < 0.05, ** *p* < 0.01 OHT vs. naïve and contralateral vs. naïve; Mann–Whitney U test. + *p* < 0.05, ++ *p* < 0.01 OHT vs. contralateral; Wilcoxon W test. GFAP: glial fibrillary acidic protein; GFAP-RA: GFAP-labeled retinal area; OHT: ocular hypertension; S: superior; I: inferior; N: nasal; T: temporal.

In both OHT and contralateral eyes, there was a significant increase in the total value of GFAP-RA with respect to that for naïve eyes (*p* < 0.01) at all the time points analyzed (1, 3, 5 and 8 days) except at 15 days, where these differences were not found (Figure 9A). When the total value of GFAP-RA was analyzed for the OHT eyes and their contralateral counterparts, significant differences were only found at Days 3 and 15 after OHT induction (both *p* < 0.05). While, at 3 days, the GFAP-RA was higher in OHT eyes; at 15 days, it was lower (Figure 9A).

In OHT eyes, the GFAP-RA variations over time showed that the greatest increase occurred at 3 days and was significant with respect to 1 day (*p* < 0.01), 5 days (*p* < 0.05) and 8 and 15 days (both *p* < 0.001) (Figure 9A). At 15 days, in OHT eyes, the GFAP-RA was significantly lower than that at the rest of the time points (1, 3, 5 and 8 days) (all *p* < 0.001) (Figure 9A). In the contralateral eyes, with the GFAP-RA time variations, an increase in GFAP-RA was found at 5 days with respect to the rest of the times; although, it did not reach statistical significance at any of the time points except at 15 days (*p* < 0.001) (Figure 9A). In turn, at 15 days, the GFAP-RA was significantly lower than at 3 and 8 days (*p* < 0.01 in both cases) (Figure 9A).

#### 3.5.2. GFAP-RA Values by Retinal Sectors

In the contralateral eyes, the sectorial analysis of GFAP-RA with respect to that in the naïve showed a significant increase in (Figure 9B):

- **Superior retina:** (i) the S-OD at 1, 3, 5 and 8 (all *p* < 0.01) days; (ii) the S-IR at 1, 3, 5, 8 (all *p* < 0.01) and 15 (*p* < 0.05) days; (iii) the S-PR at all the time points analyzed (*p* < 0.01).
- **Inferior retina:** (i) the I-OD at 1 (*p* < 0.05), 5 (*p* < 0.01) and 8 (*p* < 0.05) days; (ii) the I-IR at 1, 3 (*p* < 0.05), 5 (*p* < 0.01) and 8 (*p* < 0.05) days; (iii) the I-PR at 1, 3, 5 (*p* < 0.05) and 8 (*p* < 0.01) days.
- **Nasal retina:** (i) the N-OD at 1 (*p* < 0.01), 3 (*p* < 0.05), 5 (*p* < 0.01) and 8 (*p* < 0.05) days; (ii) the N-IR at 1, 3, 5, 8 (*p* < 0.01) and 15 (*p* < 0.05) days; (iii) the N-PR at 1, 3, 5 (*p* < 0.01) and 8 (*p* < 0.05) days.
- **Temporal retina:** (i) the T-OD only at 5 days (*p* < 0.05); (ii) the T-IR at 1, 3, 5 and 8 (*p* < 0.01) days; (iii) the T-PR at 1, 3, 5 and 8 (*p* < 0.01) days.

After sector analysis, we found that there was a significant increase in GFAP-RA in OHT eyes with respect to naïve in (Figure 9B):

- **Superior retina:** (i) the S-OD at 1, 3, 5 (all *p* < 0.01) and 8 (*p* < 0.05) days; (ii) the S-IR at 1, 3, 5 and 8 days (all *p* < 0.01); (iii) the S-PR at 1, 3, 5 and 8 (*p* < 0.01) days.
- **Inferior retina:** (i) the I-OD at 3 days (*p* < 0.01); (ii) the I-IR at 1 and 3 days (both *p* < 0.01) and at 5 and 8 days (*p* < 0.05); (iii) the I-PR at 3 (*p* < 0.01), 5 and 8 (both *p* < 0.05) days.
- **Nasal retina:** (i) the N-OD at 1 (*p* < 0.05), 3 (*p* < 0.01) and 5 (*p* < 0.05) days; (ii) the N-IR at 1, 3, 5 and 8 days (all *p* < 0.01); (iii) the N-PR at 1, 3 (both *p* < 0.01), 5 (*p* < 0.05) and 8 (*p* < 0.01) days. At 15 days, in the N-IR, we found a significant decrease in GFAP-RA (*p* < 0.05).
- **Temporal retina:** (i) the T-OD only at Day 3 (*p* < 0.01); (ii) the T-IR at 1, 3, 5 and 8 days (all *p* < 0.01); (iii) the T-PR at 1 (*p* < 0.05), 3, 5 and 8 (all *p* < 0.01) days.

The comparison between the OHT and their contralateral eyes showed a significant decrease in GFAP-RA in the OHT eyes in (Figure 9B): (i) the S-OD at 5 and 15 (*p* < 0.05) days; (ii) the S-IR at Day 8 (*p* < 0.05); (iii) the S-PR at 15 (*p* < 0.05) days; (iv) the I-IR at 15 (*p* < 0.05) days; (v) the N-OD and N-IR at 15 (*p* < 0.05) days; and (vi) the T-PR at 15 (*p* < 0.05) days. However, an increase in GFAP-RA was found in OHT eyes with respect to contralateral eyes at 3 days in the T-IR, N-IR, I-OD, I-IR and I-PR (all *p* < 0.05).

### 3.6. Intensity of GFAP Immunoreactivity at Different Time Points after OHT Induction

**Figure 10:**
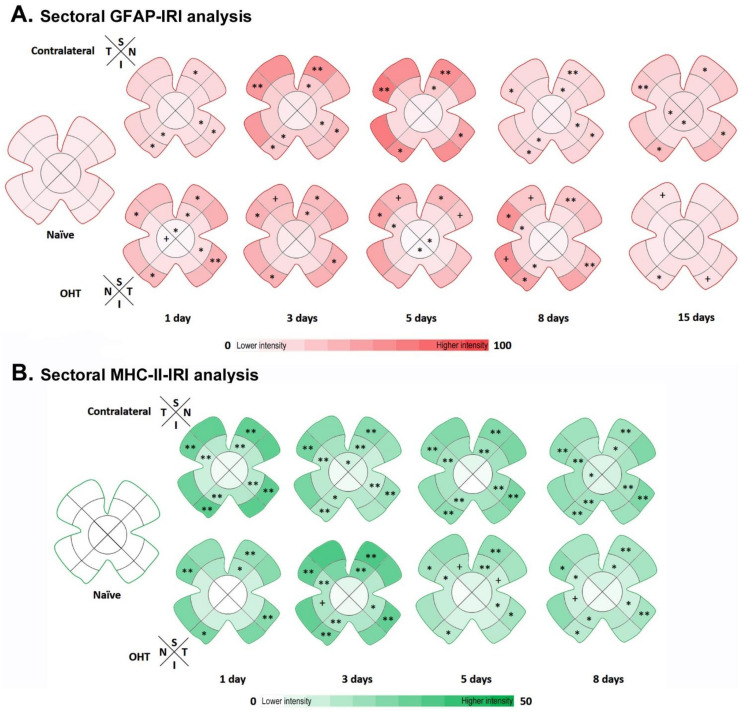
GFAP-IRI and MHC-II-IRI values at different time points after unilateral OHT laser-induction. (**A**): Colorimetric differences in the GFAP-IRI by retinal sectors in naïve eyes and at different time points after laser treatment (1, 3, 5, 8 and 15 days) in contralateral and OHT eyes. * *p* < 0.05, ** *p* < 0.01 OHT vs. naïve and contralateral vs. naïve; Mann–Whitney U test. + *p* < 0.05 OHT vs. contralateral; Wilcoxon W test. (**B**): Colorimetric differences in the MHC-II-IRI by retinal sectors in naïve eyes and at different time points after laser treatments (1, 3, 5, and 8 days) in contralateral and OHT eyes. * *p* < 0.05, ** *p* < 0.01 OHT vs. naïve and contralateral vs. naïve; Mann–Whitney U test. + *p* < 0.05 OHT vs. contralateral; Wilcoxon W test. GFAP: glial fibrillary acidic protein; GFAP-IRI: GFAP-immunoreactivity intensity; MHC-II: major histocompatibility complex class II; MHC-II-IRI: MHC-II-immunoreactivity intensity; OHT: ocular hypertension; S: superior; I: inferior; N: nasal; T: temporal.

A comparison of the contralateral and naïve eyes showed a significant increase in GFAP-IRI in (Figure 10A):

- **Superior retina:** (i) the S-IR at 3, 5 and 8 days (all *p* < 0.05); (ii) the S-PR at 1 (*p* < 0.05), 3, 5, 8 (all *p* < 0.01) and 15 (*p* < 0.05) days.
- **Inferior retina:** (i) the I-OD at 15 days (*p* < 0.05); (ii) the I-IR at 1, 3 and 8 days (all *p* < 0.05); (iii) the I-PR at all the time points analyzed (*p* < 0.05).
- **Nasal retina:** (i) the N-IR at 1, 3, and 8 days (all *p* < 0.05); (ii) the N-PR at all the time points analyzed (*p* < 0.05).
- **Temporal retina:** (i) the T-OD at 15 days (*p* < 0.05); (ii) the T-PR at 3, 5, (*p* < 0.01), 8 (*p* < 0.05) and 15 (*p* < 0.01) days.

The sectorial analysis showed that there were significant differences in the GFAP-IRI in OHT eyes with respect to naïve (Figure 10A):

- **Superior retina:** (i) a decrease in the S-OD at 1 day (*p* < 0.05); (ii) an increase in the S-IR at 1 and 3 (*p* < 0.05) days; (iii) an increase in the S-PR at 1, 3, 5 (*p* < 0.05) and 8 (*p* < 0.01) days.
- **Inferior retina:** (i) a decrease in the I-OD at 5 days (*p* < 0.05); (ii) an increase in the I-IR at 8 (*p* < 0.05) days; (iii) an increase in the I-PR at 1, 3, 8 and 15 (*p* < 0.05) days.
- **Nasal retina:** (i) an increase in the N-IR at 5 and 8 (*p* < 0.05) days; (ii) an increase in the N-PR at 1, 3, 5 and 8 (*p* < 0.05) days.
- **Temporal retina:** (i) a decrease in the T-OD at 5 days (*p* < 0.05); (ii) an increase in the T-IR at 1 day (*p* < 0.05); (iii) an increase in the T-PR at 1 day (*p* < 0.01), 3 days (*p* < 0.05) and 8 days (*p* < 0.01).

A comparative analysis for contralateral and OHT eyes showed a significant decrease in the GFAP-IRI in (Figure 10A): (i) the N-OD at 1 day (*p* < 0.05); (ii) the S-PR at 3 days (*p* < 0.01); (iii) the S-PR and T-PR at 5 days (*p* < 0.05); and (iv) the S-PR and I-PR at 15 days (both *p* < 0.05). However, an increase in GFAP-IRI was found in OHT eyes with respect to contralateral eyes in the S-PR and N-PR at 8 days (*p* < 0.05).

### 3.7. Intensity of MHC-II Immunoreactivity at Different Time Points after OHT Induction

The MHC-II-IRI was measured at all the time points after OHT induction except at 15 days, because microglial cells increased MHC-II expression at this time point, which made it impossible to separate and quantify the MHC-II-IRI of the macroglia from that of the microglial cells in both eyes (OHT and contralateral).

A comparison of contralateral and naïve eyes showed a significant increase in MHC-II-IRI in (Figure 10B):

- **Superior retina**: (i) the S-OD only at 3 days (*p* < 0.05); (ii) the S-IR at 1, 3, 5 (all *p* < 0.01) and 8 (*p* < 0.05) days; (iii) the S-PR at all the time points analyzed (all *p* < 0.01).
- **Inferior retina:** (i) I-IR at 1 (*p* < 0.01), 3 (*p* < 0.05), 5 and 8 (*p* < 0.01) days; (ii) the I-PR at all the time points analyzed (all *p* < 0.01).
- **Nasal retina:** (i) the N-IR at all the time points analyzed (all *p* < 0.01); (ii) the N-PR at all the time points analyzed (all *p* < 0.01).
- **Temporal retina:** (i) the T-OD only at 8 days (*p* < 0.05); (ii) the T-IR at all the time points analyzed (all *p* < 0.01); (iii) the T-PR at all the time points analyzed (all *p* < 0.01).

Sectorial analysis of MHC-II-IRI showed that there was a significant increase in OHT eyes with respect to naïve in (Figure 10B):

- **Superior retina:** (i) the S-IR at 1 (*p* < 0.05), 3, 5 (both *p* < 0.01) and 8 (*p* < 0.05) days; (ii) the S-PR at all the time points analyzed (all *p* < 0.01).
- **Inferior retina:** (i) the I-IR at 3 (*p* < 0.01) and 8 (*p* < 0.05) days; (ii) the I-PR at 1 (*p* < 0.05), 3 (*p* < 0.01), 5 and 8 (both *p* < 0.05) days.
- **Nasal retina:** (i) the N-IR at 3 (*p* < 0.01), 5 and 8 (both *p* < 0.05) days; (ii) the N-PR at 1, 3 (both *p* < 0.01), 5 and 8 (*p* < 0.05) days.
- **Temporal retina:** (i) the T-IR at 3, 5 and 8 (all *p* < 0.05) days; (ii) the T-PR at 1, 3 (both *p* < 0.01), 5 (*p* < 0.05) and 8 (*p* < 0.01) days.

Comparative analysis between contralateral and OHT eyes showed in OHT eyes a significant decrease in MHC-II-IRI in (Figure 10B): (i) the S-IR and T-IR at 5 days (*p* < 0.05); (ii) the N-IR at 8 days (*p* < 0.05). Nevertheless, the comparison showed a significant increase in MHC-II-IRI in OHT eyes with respect to contralateral eyes in the N-IR at 3 days (*p* < 0.05).

## 4. Discussion

This study is the first to analyze the activation of retinal macroglia (astrocytes and Müller glia) and their expression of MHC-II in a mouse model at different time points (1, 3, 5, 8 and 15 days) after unilateral OHT induction, studying both OHT and normotensive contralateral eyes, compared to naïve eyes. This study is a continuation of previous work carried out in this same experimental model of glaucoma in which the activation of microglial cells at the different time points mentioned above was analyzed [5,7,9], as well as the expression of different cytokines and chemokines [24]. This work, therefore, completes the analysis of the behavior of retinal glial cells in response to increased IOP, both in OHT eyes, and in their contralateral counterparts in this model.

The model of the unilateral laser induction of OHT is a widely used model of glaucoma [51,52,56]. In this model, the IOP is elevated 1 day after OHT induction and remains elevated until 5 days, after which it reaches normal values at 8 days [7,24]. In this model, RGC death [24,51,56] and glial cell activation occur [5,7,9,57], resembling the changes that appear in human glaucomatous neuropathy [58], and may be useful for the study of macroglial cells.

In this study, retinal whole-mounts were used for the analysis of astrocytes. Retinal astrocytes in the mouse are arranged in a plexus in the NFL–GCL parallel to the retinal surface, allowing us to perfectly differentiate their morphology and whether signs of activation such as cell hypertrophy occur. Müller glia are arranged perpendicular to the retina, so only the end feet can be observed in retinal whole-mounts. However, at the edges of the preparation, or in the areas where we make cuts to flatten the retinas in whole-mounts, when we place the coverslip, a retinal section-like effect is produced, allowing us to see the Müller cells in their full extent.

GFAP is the main intermediate filament protein in mature astrocytes [59]; therefore, an antibody against GFAP allows us to study them specifically. In addition, GFAP is a very sensitive marker of astroglial activation in response to various types of neuronal damage and is commonly used as an index of gliosis hypertrophy [60,61]. The activation of Müller glia is also related to GFAP content. As with astrocytes under pathological conditions, there is an increased expression of GFAP in Müller cells [14,15].

Macroglial cells respond via a non-specific response to different injuries to the retina such as hypoxic damage [62], light damage [63], mechanical damage [64], diabetic retinopathy [65], experimentally induced glaucoma [5,8] and others. This response, called reactive gliosis, is characterized by an increase in the number of astrocytes, in the number and length of astroglial processes, and in the size of the soma (hypertrophy), as well as the migration and upregulation of cytoskeletal components such as GFAP [12,32,33,66]. In light of this, the percentage of retinal area covered by reactive astrocytes could serve as a biomarker that could predict the health of RGCs as well as identify the risk of glaucoma-related vision loss [67].

In our study, we found that the total GFAP-RA was statistically significantly increased in both the OHT and contralateral eyes with respect to the naïve eyes at all the time points, except at 15 days after laser treatment in the OHT eye, where it was lower. The greatest increase in GFAP-RA occurred in the OHT eye at 3 days and in the contralateral eye at 5 days. Furthermore, in the contralateral eyes, the GFAP-RA was so increased that it was indistinguishable from the OHT eyes, with significant differences only at 3 days, where the area was smaller, and at 15 days, where the area was larger.

When we morphologically analyzed the astrocytes, they showed, in general, thicker somas and more secondary processes in the OHT eyes and in the contralateral eyes, compared to the naïve eyes, at all the time points after laser treatment, mainly in the peripheral and intermediate retina. This was confirmed by analyzing the GFAP-RA, which was significantly higher in the intermediate and peripheral sectors of the four quadrants (superior, inferior, nasal and temporal) of the retina. Therefore, reactive astrogliosis occurred at all the time points analyzed in both the OHT and contralateral normotensive eyes.

In both OHT and contralateral eyes, the Müller glia were GFAP+ at all the time points tested. This increase in GFAP expression has been considered a more sensitive marker of Müller glial activation, which may indicate that retinal damage is being produced [14,68].

The macrogliosis that occurs in the retina due to the increase in IOP [8] could initially be protective for retinal neurons, but could lead to neuronal damage if it becomes chronic and uncontrolled [37,39]. Initially, reactive macroglia may be involved in isolating the damage, removing dead cells and tissue debris [33]. Reactive astrocytes have been shown to phagocytose dead RGCs through a process of apoptosis or necrosis [57]. In OHT eyes from 5 days after OHT induction, we observed a decrease in GFAP-RA in the areas close to the disc. It has been shown that a decrease in GFAP-RA may be related to an increased presence of NF-200+ RGCs, indicating an abnormality in axoplasmic flow [5]. Therefore, the decrease in GFAP-RA found in our study could be related to an impairment of axonal support by macroglia, causing axons to be more vulnerable to IOP damage. In OHT retinas, an increase in BDNF expression produced by reactive macroglia has been observed [24]. This increase, which is observed from day 1 after OHT induction, could be a protective effect of RGCs against the damage induced by the increase in IOP.

The initial protective activation of macroglia may occur in the contralateral normotensive eyes, where RGC death is not yet occurring, but proinflammatory signals may be reaching them from the OHT eyes, activating macroglial cells for RGC protection [5,7,9,24]. In our study, in contralateral eyes, GFAP-RA remained elevated longer and more uniformly throughout the retina after OHT induction, than in OHT eyes, with no areas of low GFAP-RA observed in areas near the optic disc. This behavior in the absence of RGC death could support the neuroprotective role of activated macroglia in contralateral eyes.

Chronic macrogliosis can be detrimental to neurons and vessels and can cause direct or indirect damage and impair tissue repair [38]. This chronic activation of astroglia and Müller glia probably occurs in OHT eyes. In reactive macroglial cells, there is an upregulation of vascular endothelial growth factor (VEGF), leading to increased vascular permeability and even neovascularization, as observed in different retinal pathologies such as diabetic retinopathy, retinopathy of prematurity and age-related macular degeneration [69]. In previous work, in the same experimental model as in this study, we found an increase in VEGF at 1 day after OHT induction only in OHT eyes, expressed by macroglial cells [24]. This increase in VEGF could increase vascular permeability in this model by disrupting the blood–retinal barrier and allowing the entry of monocytes/macrophages from the bloodstream into the retinal tissue, as previously observed by us [9]. Molecules released by reactive macroglia can also induce neuronal death and prevent axonal regeneration. In our experimental glaucoma model, molecules released by reactive macroglia such as TNF-α and IL-1β could induce the secondary death of RGCs [24].

In the present study, we analyzed the intensity of GFAP immunoreactions at different time points after laser treatment in both the OHT eye and the contralateral eye and found variations in GFAP immunoreactions. GFAP is essential for normal astrocyte physiology and helps to maintain mechanical strength and cell shape, as well as motility and migration. During brain development and aging, changes in GFAP expression correlate with changes in astrocyte function. In addition, in vivo and in vitro studies have shown that GFAP is important for differentiation, proliferation, process formation, astrocyte–neuron interactions, vesicle trafficking and other functions [59]. In addition, GFAP has been shown to be relevant for astrogliosis processes [70].

In both OHT and contralateral eyes, a significant increase in GFAP-IRI was observed in the intermediate and peripheral retina with respect to the naïve eye, with the maximum difference in the periphery. These areas of higher GFAP-IRI coincide with areas of higher GFAP-RA and with the presence of highly activated astrocytes (with thick somas and numerous primary and secondary processes) and highly reactive Müller glia, with glial scars formed by Müller and astrocytes also observed in the OHT eyes at 5 and 8 days after laser treatment. The greater activation of astrocytes and GFAP-IRI in the peripheral retina could be due to the fact that it is the area close to the transition area of the retina with the ciliary body, and these astrocytes would play a protective role, forming a limiting tissue that would be activated more intensely in response to damage in order to protect the nervous tissue.

The mechanisms by which GFAP expression may be increased after damage could be due to the fact that the GFAP gene contains a promoter with multiple domains that can be stimulated by a variety of factors that are released by cells at the site of damage [71], such as cAMP, growth factors, hormones and cytokines (IL-1, IL-6, TNF-α, IFN-γ, TGF-β, CNT, etc.) [72]. The injection of IL-1 into the mouse brain has been shown to result in reactive astrocytes with robust processes, and the same astroglial response has also been seen upon exposure to IL-6. The ability of IL-6 to increase GFAP expression was dependent on the pSTAT3 signaling pathway [72]. In this mouse model of glaucoma in previous work, we found an increase in IL-6 expression at 1, 3 and 5 days after OHT induction in OHT eyes and at 1, 5 and 7 days in contralateral eyes. We also found an increase in IL-1β expression at 1 and 3 days after laser treatment in the contralateral eyes [24]. The increase in these cytokines could have triggered astrocyte activation in the OHT and contralateral eyes in the animals in our study.

However, in the area near the optic disc in OHT eyes, we found a decrease in GFAP-IRI reaching statistical significance at 1 day and 5 days after the laser treatment. In the morphological study, the astrocytes near the optic disc were observed to have low GFAP immunostaining, and only the soma and primary processes could be distinguished, with practically no secondary processes being observed. At 5 days, these astrocytes were also found in the intermediate retina. In the contralateral eyes, less GFAP-IRI was observed in the area near to the optic disc than in the intermediate and peripheral areas, which was more evident at 5 days than at the other time points; although, it was not significant with respect to the naïve eyes.

The downregulation of GFAP-IRI in astrocytes has also been observed in other models of glaucoma. In the rat model of glaucoma using episcleral vein cauterization, astrocytes with low immunoreactivity were also found in both OHT and contralateral eyes [73]. In a rat model of unilateral laser OHT, highly reactive astrocytes and astrocytes with thinner cell bodies and primary processes were found in the OHT eyes, with most of the secondary processes having disappeared [8]. In the same model of glaucoma in mice 15 days after the laser treatment, astrocytes with high GFAP-IRI and others with low GFAP-IRI were also observed, which only showed soma and primary processes, fundamentally [5].

The downregulation of GFAP expression has also been observed in other pathologies. In diabetic retinopathy, there is a decrease in GFAP immunoreactivity in astrocytes, and this is restored when insulin is administered, so insulin may regulate various aspects of retinal glial cell metabolism [74]. Depression also shows a decrease in GFAP immunoreactivity in the dentate gyrus, which was related to a decrease in BDNF expression [75]. Ethanol exposure also causes a transient upregulation of GFAP and then downregulation of GFAP in brain glia, resulting in astrocytes with weak GFAP staining and reduced numbers and lengths of GFAP processes [76]. Ethanol could exert these effects on astrocytes by altering cerebral blood flow, resulting in chronic ischemia [77].

The fact that most of these astrocytes with low GFAP-IRI were located in the areas proximal to the optic disc in OHT eyes in our OHT model could be because they are nearer to the area where the damage (mechanical and ischemic) is initiated by the increased IOP at the level of the optic nerve head [78]. In the cortices and hippocampi of mutant mice that do not express GFAP (GFAP−/−), reduced glutamate uptake and reduced glutamate transport activity were found [79]. In the optic nerve, it has been observed that, in GFAP−/− mutant mice, there are alterations in the blood–brain barrier (BBB); the astrocytes that form the barrier only form a single layer of astrocytic end feet, with few intermediate filaments, which makes the barrier more permeable [80]. Furthermore, in cultures of astrocytes from GFAP−/− animals, they lose the ability to induce BBB properties. Therefore, the downregulation of GFAP expression, mainly in OHT eyes, observed in astrocytes in different glaucoma models, could be related to an altered metabolic capacity and a reduced ability to maintain BRB integrity for these cells.

Astrocytes are the most numerous types of glial cell in the CNS and are involved in an important manner in the neuroinflammatory process. Macroglial and microglial cells can upregulate major histocompatibility complex (MHC) molecules, allowing them to participate in adaptive immunity [81]. MHC-II, which allows antigen presentation to CD4+ helper T cells, is expressed by professional antigen-presenting cells (APCs) such as dendritic cells, B cells and macrophages [82]. However, in the CNS, due to their myeloid origin, microglia have been considered the main APCs, but several studies have indicated that microglia are often not the main antigen presenters [83,84], and it has been shown that astrocytes play an important role in this function. When astrocytes are activated, they express MHC-II and its co-stimulatory molecules on their surfaces to stimulate T cells in the CNS [85].

To study the activation of macroglia with OHT, we analyzed MHC-II-IRI at different time points after laser treatment. We found an increase in MHC-II-IRI in both OHT eyes and their normotensive contralateral counterparts at all the time points analyzed with respect to naïve eyes. The areas where this MHC-II-IRI was the highest were in the intermediate and peripheral retina, which coincides with the location of the most reactive astrocytes and the highest GFAP-IRI. In the areas where astrocytes with low GFAP immunostaining were observed (areas close to the optic disc), virtually no significant changes in MHC-II-IRI with respect to that in naïve eyes were observed. This would indicate a low capacity to present the antigen.

In human glaucoma, the expression of MHC-II (specifically HLA-DR) by astrocytes and persistently activated microglia has also been observed [86]. In other studies using the same OHT model as in the present work, at 15 days after laser treatment, MHC-II expression was observed in astrocytes, Müller glia and microglia in both OHT and contralateral eyes [5,9,57]. In our study, we found that, at 1 day after laser treatment, the reactive astrocytes began to express MHC-II, whereas in reactive Müller glia, this expression was evident from Day 5. This occurs in both OHT and contralateral eyes. At 15 days after laser treatment, the pattern of MHC-II expression changes; the expression being more evident in Müller glia in OHT eyes and in astrocytes in contralateral eyes. This last observation agrees with the study of Gallego et al. (2012) [5], in which, at this point, as we could observe, there was this same pattern of MHC-II expression by astrocytes and Müller glia and, additionally, a high expression of MHC-II by microglia.

Astrocyte and Müller glia activation as well as MHC-II expression is observed in both OHT eyes and their normotensive contralateral counterparts, being similarly intense in both eyes. The macroglial activation and MHC-II expression in the contralateral eye could be due to the bilateral alteration of retinal immunoprivilege. The damage generated in the OHT eye could be responsible for the overall broad inflammatory response that would reach the contralateral eye, increasing MHC-II in both eyes [87].

It has been proposed that interferon-γ (IFN-γ) (a proinflammatory cytokine) could be involved in the expression of MHC-II by astrocytes and, thus, in their activation [88]. This process has been demonstrated in primary cultures of rodent astrocytes [89] and in human astrocytes [90]. IFN-γ produced by immune cells, such as microglia in the CNS, would bind to the receptor located on astrocytes (IFN-γ-R1) and stimulate the JAK/STAT signaling cascade, with a consequent transcriptional response for MHC-II expression [91]. MHC-II has been observed to be transferred between astrocytes through tunnelling nanotubes, suggesting a mechanism for the spread of inflammation in the brain [90]. This pathway could also be involved in the transmission of the inflammatory process from the OHT eye to the contralateral eye. In our mouse OHT model, in a previous study [24], we observed IFN-γ expression in the retinas of OHT and contralateral eyes, and this cytokine may be involved in MHC-II macroglial expression in our study.

In neurodegenerative processes, such as glaucoma, the microglial cell is the main mediator in the neuroinflammatory response; however, astrocytes can act synergistically with microglia, promoting neuroprotection or chronic neuroinflammation [40]. Astrocyte–microglia interaction is necessary for astrocytes to support neuronal survival after damage [92,93].

In our mouse OHT model, microglia constitutes the first line of defense, responding early on to increased IOP by releasing IL-6 [24]. This cytokine can activate astrocytes, which release factors such as VEGF, BDNF, TNF-α and IL-1β that can modulate the inflammatory process [24]. This has been observed in other pathologies, where microglia would first be activated and recruited to the site of damage for cellular debris removal and, secondarily, activate astrocytes, releasing inflammatory factors that contribute to the chronic activation of the microglia [92,94].

In the present work, we found that, in general, although changes related to macroglial activation (morphological changes, GFAP-RA, GFAP-IRI and MHC-II-IRI) occurred at all the time points, these could be considered more intense at Days 3 and 5. This coincides with previous work in this same experimental model, in which we found a greater activation of microglial cells at Days 3 and 5 and a downregulation of P2RY12 expression in these cells [7], in addition to observing the main changes in cytokine and myokine expression after OHT induction [24]. All this would indicate a greater inflammatory process at these time points after OHT induction in this experimental model of laser-induced glaucoma.

## 5. Conclusions

In this study, we observed that, after the induction of unilateral OHT, macroglial (astrocyte and Müller) activation occurred and was observed at all the time points (1, 3, 5, 8 and 15 days after laser treatment), with morphological changes (GFAP-RA) as well as changes in the expression of GFAP and MHC-II. The highest activation of macroglial cells occurred in the intermediate and peripheral retina; however, astrocytes with low GFAP and MHC-II expression were found in the retina proximal to the optic disc. Although macroglial activation was observed at all the time points after laser treatment, it was slightly more intense at 3 and 5 days, coinciding with the pattern of activation observed in previous studies, in which the main changes in microglial activation, as well as in the expression of several cytokines and myokines related to the inflammatory process in glaucoma, occurred at 3 and 5 days after OHT induction. These macroglial changes were observed in both OHT eyes and their normotensive contralateral counterparts, such that activation in the contralateral eye could be triggered by immune signals derived from the OHT eyes. Therefore, in the unilateral laser-induced OHT model, a bilateral inflammatory process maintained over time in which microglia, astrocytes and Müller glia are involved occurs.

## Data Availability

The data supporting the findings of this study are available from the corresponding author upon request.

## Supplementary Materials

The following supporting information can be downloaded at: www.mdpi.com/article/10.3390/biomedicines10050939/s1. Figure S1. Intraocular pressure values after unilateral OHT laser-induction. Figure S2. Retinal whole-mount. GFAP immunostaining in naïve eyes and at different time points after OHT induction in OHT and contralateral eyes. Figure S3. Retinal whole-mount. Double immunostaining for GFAP (red) and MHC-II (green) in naïve eyes. Figure S4. Retinal whole-mount. Double immunostaining for GFAP (red) and MHC-II (green) at different time points after OHT induction in contralateral eyes in the retina near to the optic disc. Figure S5. Retinal whole-mount. Double immunostaining for GFAP (red) and MHC-II (green) at different time points after OHT induction in OHT eyes in the retina near to the optic disc. Figure S6. Retinal whole-mount. GFAP immunostaining in OHT eyes. Glial scar.
