# Supplementary material for: Retinal Changes in Astrocytes and Müller Glia in a Mouse Model of Laser-Induced Glaucoma: A Time-Course Study
Source: Biomedicines. 2022 Apr 19;10(5):939. doi: 10.3390/biomedicines10050939 (PMC9138377; doi:10.3390/biomedicines10050939)
Supplement: Supplementary file 1 [file biomedicines-10-00939-s001.zip › Supplementary Material reviewers.pdf]

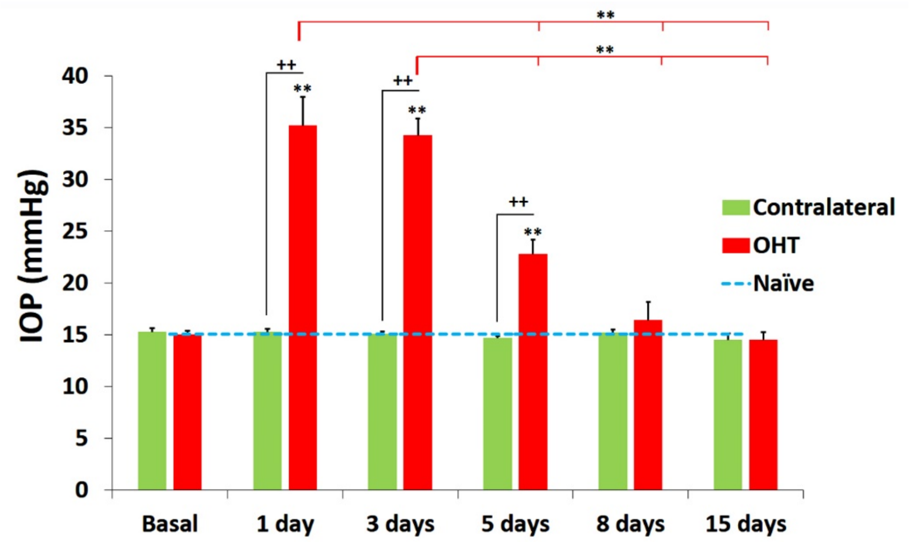

Figure S1. Intraocular pressure values after unilateral OHT laser-induction. Histogram shows the mean values of IOP (±SD) at different time points after laser treatment (baseline and 1, 3, 5, 8 and 15 days) in naïve, contralateral and OHT eyes. Dashed line represents IOP measurements in naïve eyes. In laser-treated OHT eyes, the IOP increase at 1 day remained statistically significantly elevated until 5 days and reached normal values at 8 days after laser treatment. In contralateral eyes, IOP values similar to those of naïve eyes were observed at all time points. **p<0.01 OHT vs. naïve, Mann–Whitney U test; ++p<0.05 OHT vs. contralateral, Wilcoxon W test. OHT variation over time: ANOVA Bonferroni test.

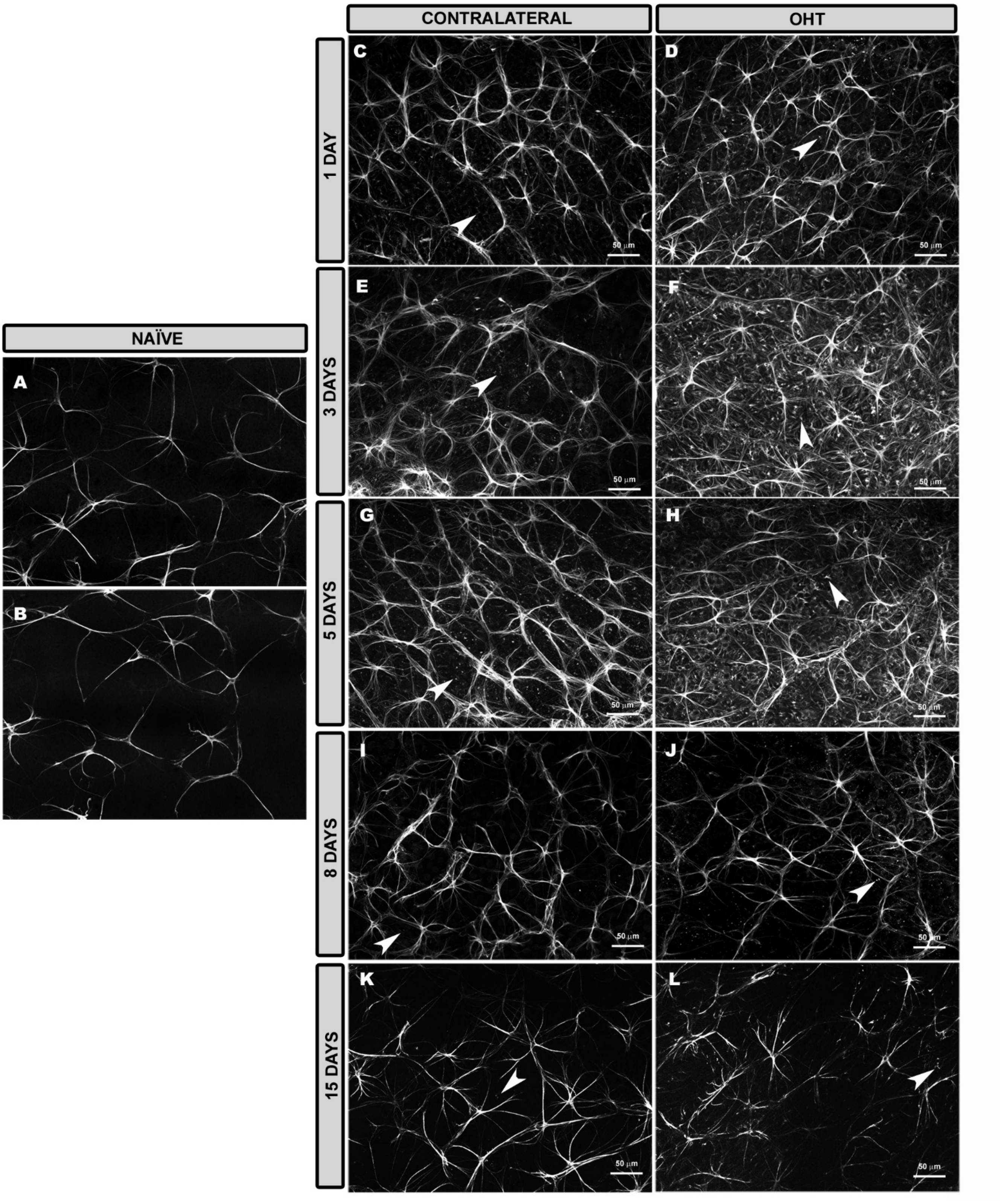

Figure S2. Retinal whole-mount. GFAP immunostaining in naïve eyes and at different time points after OHT induction in OHT and contralateral eyes. Overview of retinal macroglial cells plexus (astrocytes and Müller glia) in intermediate retina in naïve eyes (A, B) and at different time-points after laser (1, 3, 5, 8 and 15 days) in contralateral (C, E, G, I, K) and OHT (D, F, H, J, L) eyes. Astrocytes were reactive in 1, 3, 5 and 8 days after laser both in OHT (D, F, H, J) and in contralateral (C, E, G, I)

compared to naïve (A, B), showing thicker cell bodies and numerous processes, therefore a higher GFAP-RA. At 15 days after laser treatment in contralateral eyes (K) astrocytes were slightly more reactive than naïve, however in OHT eyes (L) we observed less thickened and immunoreactive astrocytes than naïve eyes in some areas alternating with more immunoreactive astrocytes, resulting in less GFAP-RA. The end feet of the Müller cells (arrowhead) showed a punctate GFAP+ between astrocytes throughout the retina it was more evident at 3 (F) and 5 (H) days in OHT eyes. GFAP: glial fibrillary acidic protein; OHT: ocular hypertension.

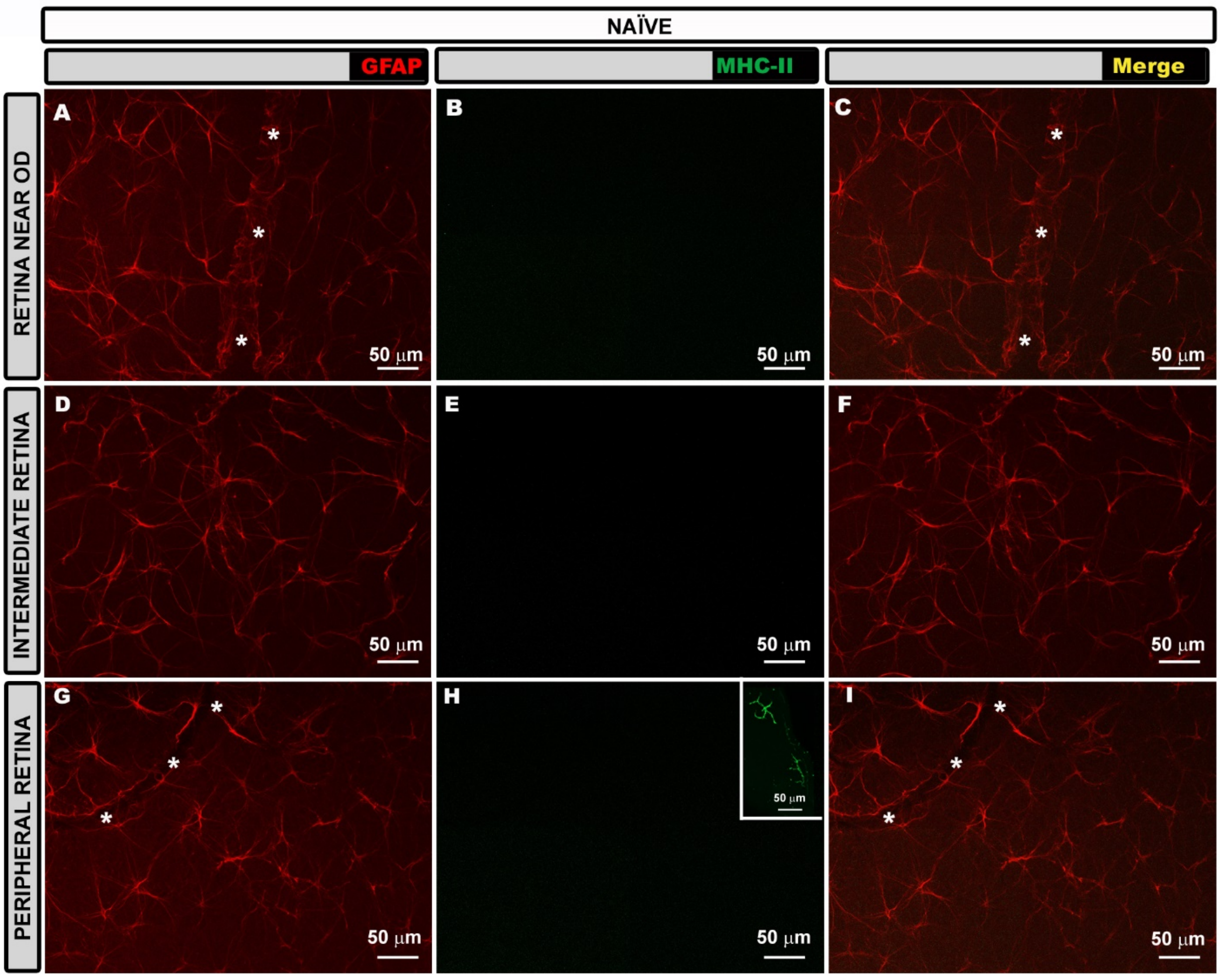

Figure S3. Retinal whole-mount. Double immunostaining for GFAP (red) and MHC-II (green) in naïve eyes. In naïve eyes, no MHC-II immunoreactivity was observed in any retinal sector such as the retina near optic disc (B,C), the intermediate retina (E,F) and the peripheral retina (H,I). However, dendritic cells showed intense MHC-II immunoreactivity (insert in H). Asterisks mark blood vessels. GFAP: glial fibrillary acidic protein; MHC-II: major histocompatibility complex class II; OHT: ocular hypertension; OD: optic disc.

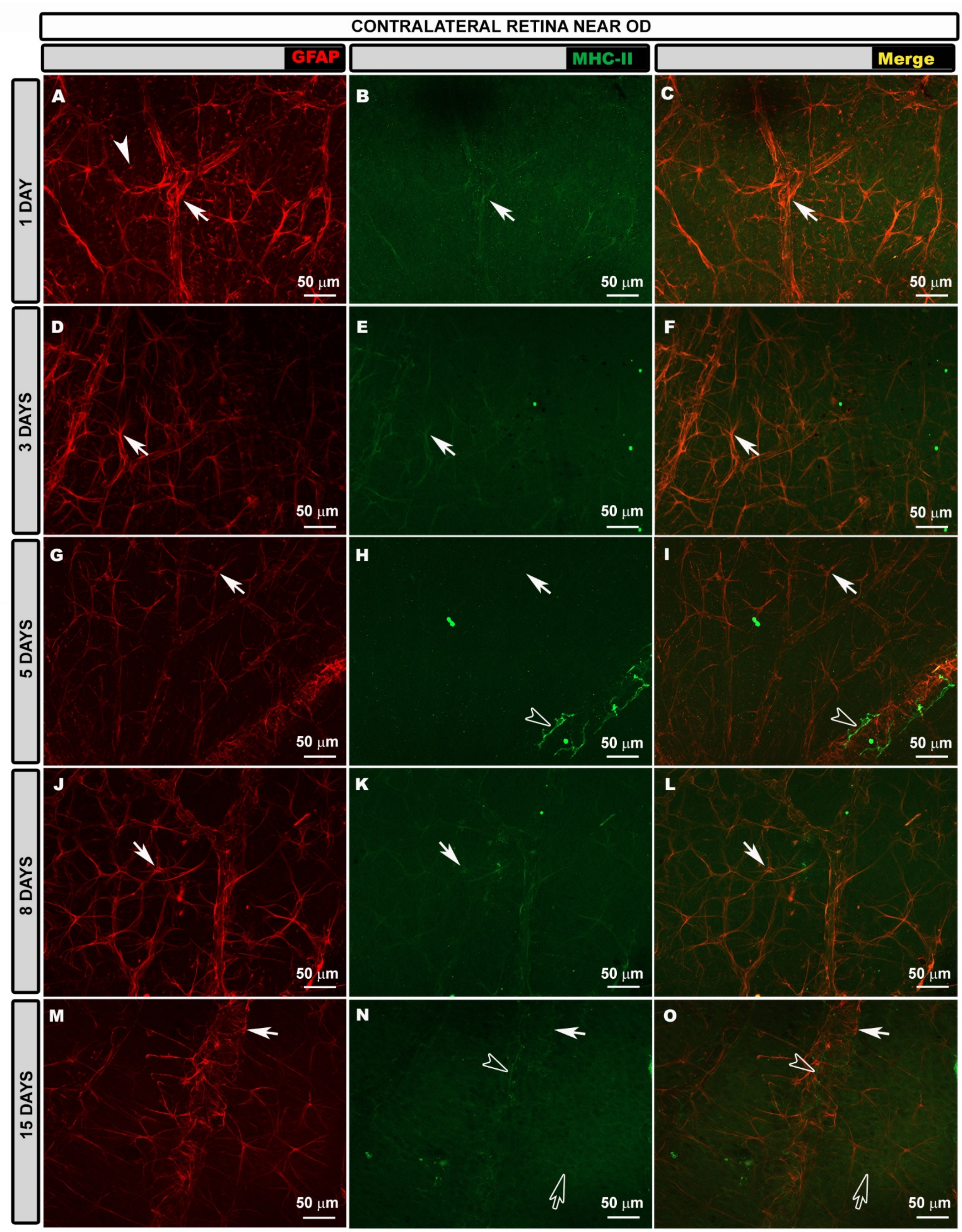

CONTRALATERAL RETINA NEAR OD
GFAP
MHC-II
Merge
1 DAY
3 DAYS
5 DAYS
8 DAYS
15 DAYS
A
B
C
D
E
F
G
H
I
J
K
L
M
N
O
50 µm

Figure S4. Retinal whole-mount. Double immunostaining for GFAP (red) and MHC-II (green) at different time points after OHT induction in contralateral eyes in the retina near to the optic disc. At the OD level in contralateral eyes, MHC-II expression observed in astrocytes (arrow) was very low at all time points (1 (B,C); 3 (E,F); 5 (H,I); 8 (K,L); and 15 (N,O) days) after laser treatment. Images show perivascular microglia (hollow arrowhead) with high MHC-II expression (H,I,N,O). At 15 days after laser treatment, microglia (hollow arrow) also showed MHC-II (N,O) immuno-labeling. GFAP: glial fibrillary acidic protein; MHC-II: major histocompatibility complex class II; OHT: ocular hypertension; OD: optic disc.

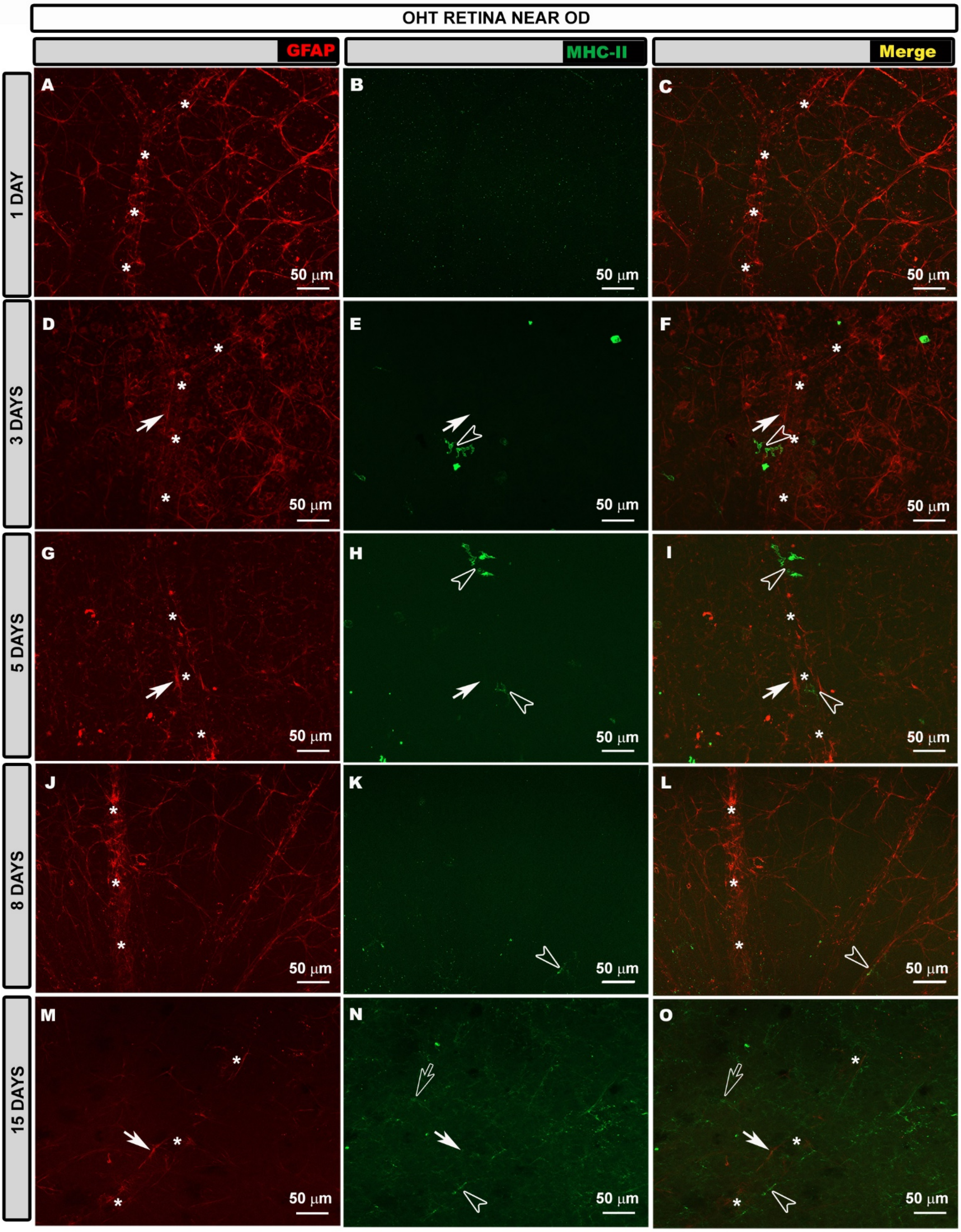

OHT RETINA NEAR OD
GFAP
MHC-II
Merge
1 DAY
3 DAYS
5 DAYS
8 DAYS
15 DAYS
A
B
C
D
E
F
G
H
I
J
K
L
M
N
O
50 µm

Figure S5. Retinal whole-mount. Double immunostaining for GFAP (red) and MHC-II (green) at different time points after OHT induction in OHT eyes in the retina near to the optic disc. In the vicinity of the optic disc, the expression of MHC-II in astrocytes (arrow) was very low and was only observed in perivascular astrocytes at all time points (1 (B,C); 3 (E,F); 5 (H,I); 8 (K,L); and 15 (N,O) days) after laser treatment. At 15 days after laser treatment (N,O), the microglia (hollow arrow) expressed MHC-II. The hollow arrowhead points to the dendritic cells. Asterisks mark blood vessels. GFAP: glial fibrillary acidic protein; MHC-II: major histocompatibility complex class II; OHT: ocular hypertension; OD: optic disc.

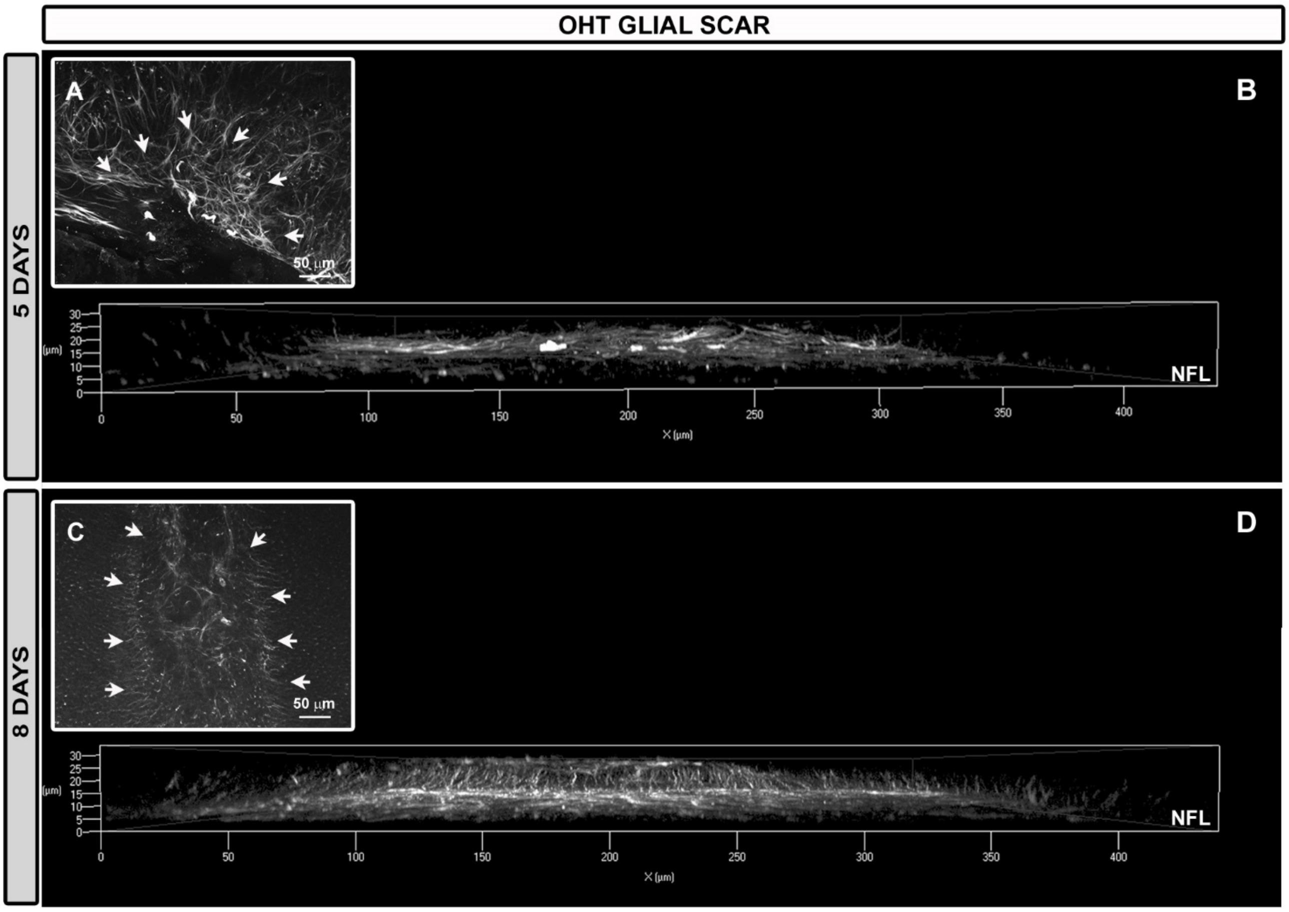

Figure S6. Retinal whole-mount. GFAP immunostaining in OHT eyes. Glial scar. In peripheral retina, at 5 (A, B) and 8 (C, D) days, reactive Müller glia and astrocytes were observed reacting to injury establishing glial scar. Retinal whole-mount immunolabeled with GFAP (A, C) corresponding to areas, in peripheral retina, where a glial scar was observed. Cut-view analysis in the YZ plane (B, D) of these glial scar. NFL: nerve fiber layer; GFAP: glial fibrillary acidic protein; OHT: ocular hypertension. Arrows delimited the extension of the glial scar (A, C).
